# Temporal merging into pitch with click train in the macaque auditory cortex

**DOI:** 10.1093/nsr/nwaf026

**Published:** 2025-01-22

**Authors:** Peirun Song, Haoxuan Xu, Hangting Ye, Xinyu Du, Yuying Zhai, Xuehui Bao, Qianyue Huang, Ishrat Mehmood, Hisashi Tanigawa, Wanqiu Niu, Zhiyi Tu, Pei Chen, Tingting Zhang, Xuan Zhao, Xiongjie Yu

**Affiliations:** Department of Anesthesia, Women's Hospital, Zhejiang University School of Medicine, Hangzhou 310006, China; Zhejiang Provincial Key Laboratory of Precision Diagnosis and Therapy for Major Gynecological Diseases, Women's Hospital, Zhejiang University School of Medicine, Hangzhou 310029, China; College of Biomedical Engineering and Instrument Science, Zhejiang University, Hangzhou 310058, China; Department of Anesthesia, Women's Hospital, Zhejiang University School of Medicine, Hangzhou 310006, China; Zhejiang Provincial Key Laboratory of Precision Diagnosis and Therapy for Major Gynecological Diseases, Women's Hospital, Zhejiang University School of Medicine, Hangzhou 310029, China; College of Biomedical Engineering and Instrument Science, Zhejiang University, Hangzhou 310058, China; Department of Anesthesia, Women's Hospital, Zhejiang University School of Medicine, Hangzhou 310006, China; Department of Anesthesia, Women's Hospital, Zhejiang University School of Medicine, Hangzhou 310006, China; Department of Anesthesia, Women's Hospital, Zhejiang University School of Medicine, Hangzhou 310006, China; Zhejiang Provincial Key Laboratory of Precision Diagnosis and Therapy for Major Gynecological Diseases, Women's Hospital, Zhejiang University School of Medicine, Hangzhou 310029, China; College of Biomedical Engineering and Instrument Science, Zhejiang University, Hangzhou 310058, China; College of Biomedical Engineering and Instrument Science, Zhejiang University, Hangzhou 310058, China; College of Biomedical Engineering and Instrument Science, Zhejiang University, Hangzhou 310058, China; College of Biomedical Engineering and Instrument Science, Zhejiang University, Hangzhou 310058, China; Department of Anesthesiology, Shanghai Tenth People's Hospital, Tongji University School of Medicine, Shanghai 200072, China; Department of Anesthesiology, Shanghai Tenth People's Hospital, Tongji University School of Medicine, Shanghai 200072, China; Department of Anesthesiology, Shanghai Tenth People's Hospital, Tongji University School of Medicine, Shanghai 200072, China; Department of Anesthesiology, Shanghai Tenth People's Hospital, Tongji University School of Medicine, Shanghai 200072, China; Department of Anesthesiology, Shanghai Tenth People's Hospital, Tongji University School of Medicine, Shanghai 200072, China; Department of Anesthesia, Women's Hospital, Zhejiang University School of Medicine, Hangzhou 310006, China; Zhejiang Provincial Key Laboratory of Precision Diagnosis and Therapy for Major Gynecological Diseases, Women's Hospital, Zhejiang University School of Medicine, Hangzhou 310029, China; College of Biomedical Engineering and Instrument Science, Zhejiang University, Hangzhou 310058, China; Department of Anesthesiology, Shanghai Tenth People's Hospital, Tongji University School of Medicine, Shanghai 200072, China

**Keywords:** temporal merging, auditory cortex, macaque monkey, click train, biomarker

## Abstract

Temporal integration stands as a cornerstone of auditory perception, yet its underlying neural mechanisms have remained relatively elusive. The intricate process by which discrete auditory stimuli integrate into cohesive perception is defined as ‘temporal merging’ in this study. We use a paradigm—the transitional click train—to probe the intricacies of temporal merging within the auditory cortex. The protocol underscores a robust change response in an adapted auditory cortex upon introducing a perceptual switch between distinct pitches. Our findings delineate four pivotal determinants that modulate this change response: train duration, inter-click interval (ICI) length, ICI contrast and train regularity. Comparative analyses between the auditory cortex and the medial geniculate body underscore a cortical origin for this temporal merging, diverging from traditional thalamic inputs. Furthermore, the clinical potential of the change response is explored, demonstrating its promise as a biomarker in anesthesia monitoring and psychiatric conditions. Collectively, this research elucidates the neuronal underpinnings of temporal integration in auditory perception, provides initial evidence for the neuronal mechanisms underlying pitch perception with click trains and introduces a potent paradigm with vast clinical implications.

## INTRODUCTION

Sound, inherently temporal, requires temporal integration for auditory perception. While traditional auditory research has primarily focused on the frequency domain, guided by the tonotopic organization of the auditory system, which processes distinct frequencies separately along the auditory pathway [1], it is paramount to acknowledge the fundamental role of the temporal dimension in auditory processing. Elements such as rhythm, timing and the intricate recognition of complex sound patterns heavily rely on temporal information [2]. This temporal dimension forms the bedrock of crucial aspects of speech and music perception, as well as the discrimination of environmental sounds [3]. Temporal coherence—a key principle in auditory scene analysis—plays a critical role in grouping acoustic elements that change synchronously, enabling the formation of coherent auditory objects and the segregation of complex auditory scenes into distinct perceptual streams [4]. This mechanism underscores how the brain utilizes temporal dynamics to organize auditory inputs into structured representations of the auditory world. In humans, temporal integration occurs across various timescales [5], as evidenced by studies in oral language processing in which the brain synchronizes with syllables, phrases and sentences at different temporal scales [6]. Even within a single word, the brain must integrate information spanning tens to hundreds of milliseconds [7]. However, there is a gap in our understanding of temporal integration at the neuronal level, to our knowledge, as no direct signal related to the grouping of individual sounds into a unified auditory perception has been identified in the current literature. The process of how successive auditory stimulations with small temporal gaps merge into a coherent auditory experience remains underexplored.

To address this question, we must create sounds that comprise distinct components and differentiate the holistic response from the responses to the constituent elements. As these two kinds of responses typically overlap, direct differentiation is challenging. Moreover, prior evidence suggests that the brain combines small auditory elements into a single sound using spectrotemporal modulation filtering [8,9], further complicating the understanding of temporal integration. Click trains, which consist of identical pulses that differ in temporal information, offer an ideal stimulus for investigating temporal processing in the auditory system. While click trains have been widely employed in auditory research, most studies have focused primarily on encoding click rates [10–13] rather than exploring holistic temporal integration. For example, studies such as those by Lu *et al.* (2001) and Bartlett and Wang (2007) [10,11] examined how neurons encode click rates through temporal or rate coding in neuronal spikes, capturing only a segmented view of auditory processing. This approach does not address how successive auditory events are integrated into a unified percept. Notably, a regular click train would be perceived as a pitch when the inter-click interval (ICI) is <33 ms [14], though the neuronal mechanisms underlying pitch perception from click trains have yet to be thoroughly investigated.

Our empirical data substantiate this hypothesis, revealing that the auditory cortex indeed exhibits a change response when exposed to the transitional click train. Further experiments have confirmed that this change response is inextricably linked to temporal integration, serving as a key indicator of the holistic representation of the click train as a pitch. These findings provide neuronal evidence for a temporal integration mechanism underlying pitch perception in the click train.

## RESULTS

The human brain typically integrates individual sounds into a cohesive perception when the gap between these sounds is exceedingly small. For instance, with click trains, humans are unable to perceive the gaps when the ICI is <29.6 ms (Fig. S1), suggesting the occurrence of temporal integration, in which individual clicks merge into a unified auditory pitch perception at the psychophysical level. This process, in which sounds with small auditory gaps integrate into a singular auditory experience, is termed ‘temporal merging’. To explore the signal that represents this temporal integration, we conducted recordings of the electrocorticography (ECoG) signal in the auditory cortexes (ACs) of two rhesus monkeys (Fig. S2A). It was observed that the AC is unable to distinguish an individual click when the ICI of the click train is extremely short, such as 4 ms (Fig. S2B and C). While prior research that employed click trains predominantly focused on the encoding of the rate of the click train [10–13], it is crucial to acknowledge that such studies have encountered constraints in their capacity to provide insights into the representation of temporal integration, particularly for the neural representation of temporal pitch associated with click trains.

### Temporal merging into pitch with click trains

**Figure 1.**
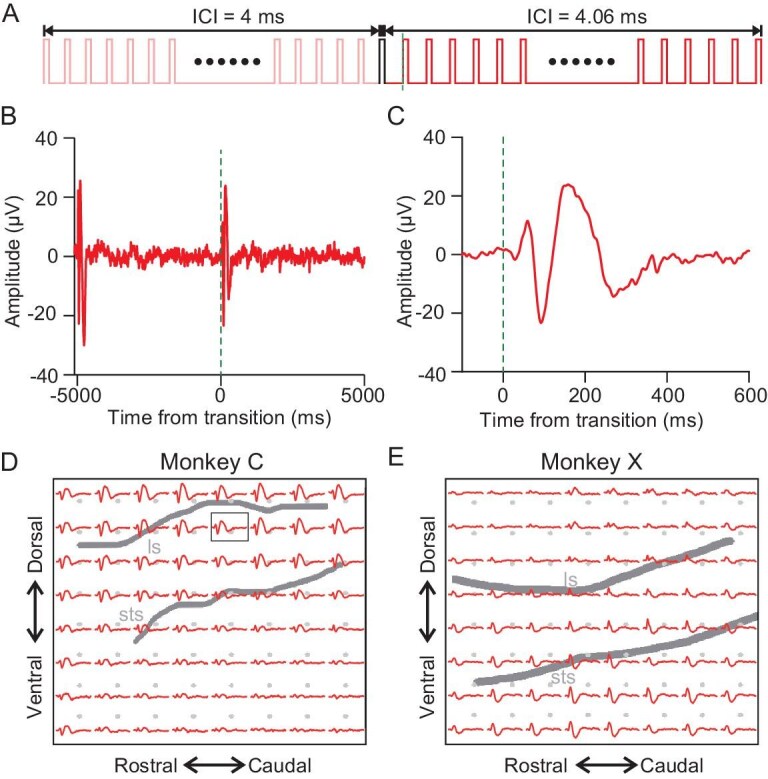
Change response in the transitional train. (A) Structure of the transitional train. Each block consists of a 5-second regular click train with 4-ms ICI followed by another 5-second regular click train with 4.06-ms ICI. The transitional train is labeled as Reg_4__–4__.06_. A common pulse is shared by both trains and the dashed line marks the transition point. (B) Responses of one example channel to Reg_4__–4__.06_. The dashed line shows the transition point time. (C) Magnified view from (B) showing the window from –100 to 600 ms relative to the transition point in the transitional train. (D) and (E) ECoG recording reveals the responses to Reg_4__–4__.06_ for all 64 channels in two monkeys. Each subplot corresponds to one recording site with a window from 0 to 600 ms relative to the transition point, as indicated by the dashed lines. Bold lines in the background highlight two key anatomical landmarks in the cortex: the lateral sulcus (ls) and the superior temporal sulcus (sts). The box indicates the location of the example channel in (B) and (C).

To investigate whether this temporal merging pitch carries any meaningful information, we used a protocol called a transitional train. We formed a transitional train by concatenating two 5-s regular click trains with different ICIs: one with 4 ms and the other with 4.06 ms (Fig. 1A). We named the transitional trains as Reg_4__–4__.06_. The Supplementary file contains the audio sounds. Consistently with previous research [15,16], the rationale behind this set-up is that, if one click train with constant ICI merges into a distinctive pitch, then the transitional train should elicit a change response in the AC, as it represents a switch between two different pitches. Figure 1B illustrates the response of an example ECoG site to Reg_4__–4__.06_. The trace shows an onset response at the start of the train and ceases to respond to the following clicks due to adaptation; however, at the transitional point of a new click train with another ICI introduced, the adapted channel showed an obvious response (Fig. 1B). The enlarged figure illustrates a clear change response within the first 600 ms after the transitional point (Fig. 1C). Figure 1D and E portrays the responses for all 64 recording sites in both monkeys’ AC. Most sites showed significant change responses for Reg_4__–4__.06_ in both monkeys (Fig. 1D and E). Comparable responses were also observed under the Reg_4.06–4_ condition, as depicted in Fig. S3. Notably, the latency of the change response was significantly prolonged in comparison with the onset response across data sets from both monkeys (two-way analysis of variance (ANOVA), *F*(1, 252) = 700.1, *P* < 0.001, Fig. S4B), as evidenced in Fig. S4.

To assess whether the generation of the change response was linked to the transition of the corresponding frequencies in the click train, a stimulus (Tone_250__–2__46_) in which the frequency shifted from 250 to 246 Hz of pure tone was designed and compared with Reg_4__–4__.06_ (Fig. S5A). Surprisingly, even though the responses to both stimuli were normalized (see Methods) based on the onset responses to ensure an equal level of onset response, no obvious change response was elicited by Tone_250__–2__46_, in contrast to the clear response observed for Reg_4__–4__.06_ (Fig. S5B–G) in both monkeys. This indicated that the change response was not primarily induced by repetition-rate-related frequency transitions.

In an effort to distinguish whether the change response was underpinned by temporal integration or was merely a response to transient changes within the transitional click trains—specifically, changes in the click intervals or sound pressure level (SPL)—two experimental paradigms were designed. Firstly, a stimulus (Reg_4__–4__.06–4_) featuring a 4.06-ms interval within a click train with 4-ms ICI was introduced and its response was compared to that of Reg_4__–4__.06_ (Fig. S6A). Interestingly, no obvious change response was observed for Reg_4__–4__.06–4_ whereas a clear response was evident for Reg_4__–4__.06_ (Fig. S6B–G). This suggested that the change response necessitated temporal integration rather than responding solely to transient alterations in the click interval. In a follow-up experiment, we equalized the SPL between two click trains (see Methods) to ascertain whether the SPL decrease due to transition from an ICI of 4 to 4.06 ms initiated the change response (Fig. S7A). Both the illustrative example and the data from two ECoG arrays manifested pronounced change responses (Fig. S7B–E). These findings consolidate the idea that the change response is anchored in temporal integration rather than the transient stimulus change. Subsequent psychophysical experiments reinforced this, demonstrating that click trains with disparate ICIs are perceived as separate auditory entities (Fig. S8). Thus, the change response seemingly corresponds to the perceptual shift between temporal pitches, coalesced through temporal integration.

**Figure 2.**
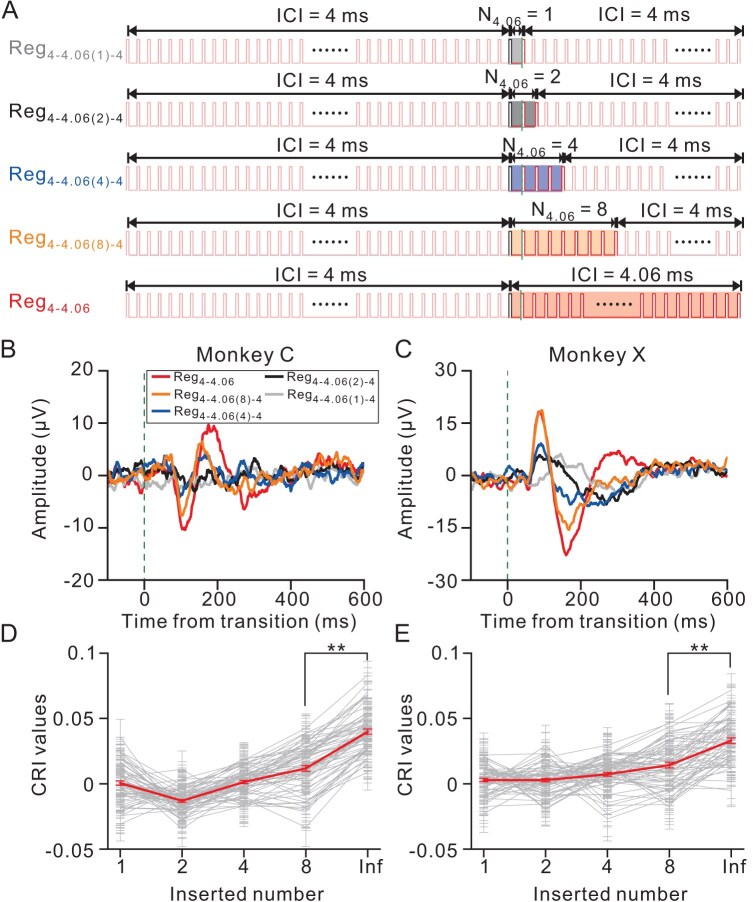
Evaluating temporal merging via interval insertion. (A) This figure outlines the structure of the stimulation used to assess temporal merging. Four distinct interval numbers (one, two, four and eight intervals of 4.06 ms) were inserted at the 2-second mark within a 3-second regular click train maintaining a constant ICI of 4 ms (shown in the top four rows). These click trains are designated as Reg_4__–__4.06(_*_n_*_)__–__4_, where ‘*n*’ represents the number of intervals inserted. For control, the bottom row illustrates Reg_4__–__4.06_, composed of a 2-second click train with 4-ms ICI, followed by a 1-second train with 4.06-ms ICI. These five patterns were presented in a randomized order. Example channels from (B) Monkey C and (C) Monkey X showing the change responses to click trains with different sets of inserted intervals compared with the control. (D) and (E) CRI values as a function of the number of inserted 4.06-ms intervals for Monkey C (left panel) and Monkey X (right panel). Each gray line represents one recording site and the bold line represents the average.

To delve deeper into the properties of the temporal merging, a systematic exploration was undertaken that involved the insertion of one, two, four and eight intervals (each with an interval of 4.06 ms) into a click train with 4-ms ICI. The responses were then compared to those of Reg_4__–4__.06_ (Fig. 2A). Strikingly, when the root mean square within the 300-ms windows was compared before and after insertion, no discernible responses were detected for either one or two inserted intervals in two example channels from each monkey, respectively (*P* > 0.05, *t*-test, Fig. 2B and C). This suggests that the change response is likely driven by temporal integration. The change response only manifested after the insertion of four intervals in the example channel from monkey X and eight intervals in the example channel from monkey C (*P* < 0.05, *t*-test). When population analysis was conducted, the Change Response Index (CRI), which is a metric that characterizes the response magnitude (see Methods), progressively resembled the response of Reg_4__–4__.06_ as the number of inserted intervals increased. However, even with eight inserted intervals, the response did not match the magnitude of the response elicited by Reg_4__–4__.06_ (*P* < 0.001 for both monkeys, ANOVA with post-hoc test, Fig. 2D and E). This indicated that the change response observed in Reg_4__–4__.06_ necessitated the integration of more than eight intervals.

### The effect of train duration

**Figure 3.**
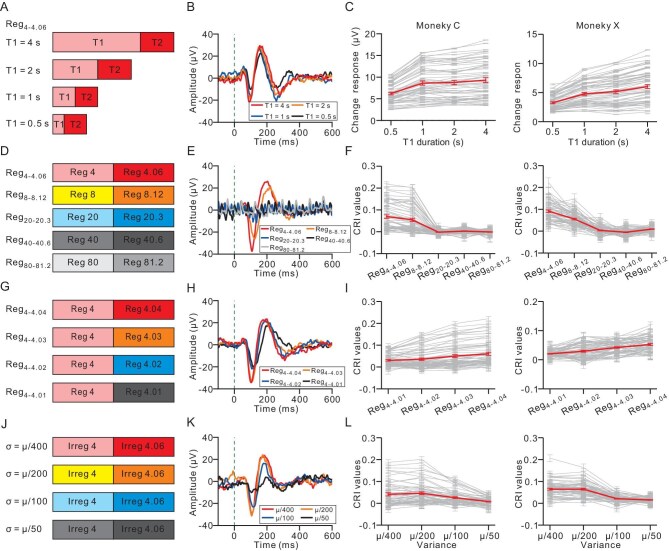
The effect of four factors on the change response in the transitional train. (A–C) The effect of the duration. (A) Stimulation set-up. Four levels of duration were chosen for the preceding click train in the transitional train: 4, 2, 1 and 0.5 s. The latter click train had the same duration (2 s). The ICI of the preceding and the latter click trains were 4 and 4.06 ms, respectively. The four blocks were presented randomly. (B) An example channel showing the change responses in the transitional trains for the four durations of the preceding click train. (C) CRI values as a function of the duration of the preceding click train for Monkey C (left panel) and Monkey X (right panel). Each light line represents one recording site and the bold line represents the average. Error bars denote the standard error (SE) of the mean. (D and E) The effect of the click interval length. (D) Stimulation set-up. Five combinations of click interval were chosen in the transitional trains: 4–4.06, 8–8.12, 20–20.3, 40–40.6 and 80–81.2 ms. The five blocks were presented randomly. (E) One example channel showing the change response in the transitional trains for the five interval combinations. (F) CRI values as a function of the interval combination for Monkey C (left panel) and Monkey X (right panel). Each light line represents one recording site and the bold line represents the average. (G–I) The effect of the interval difference. (G) Stimulation set-up. Four levels of interval difference were chosen for the transitional trains: 4–4.01, 4–4.02, 4–4.03 and 4–4.04 ms. The four blocks were presented randomly. (H) An example channel showing the change response in the transitional trains for the four interval differences. (I) CRI values as a function of the interval difference for Monkey C (left panel) and Monkey X (right panel). Each light line represents one recording site and the bold line represents the average. (J–L) The effect of the ICI variance. (J) Stimulation set-up. Four levels of ICI variance were chosen for the irregular combinations in Irreg_4-4.06_: *µ*/400, *µ*/200, *µ*/100 and *µ*/50 (*µ* = 4 or 4.06 ms). The four blocks were presented randomly. (K) An example channel showing the change response in the irregular combinations for the four levels of ICI variance. (L) CRI values as a function of the ICI variance for Monkey C (left panel) and Monkey X (right panel). Each light line represents one recording site and the bold line represents the average.

Having established the reliance of the change response on temporal integration, our investigation proceeded to delineate the minimal and maximal durations necessary for the temporal merging of auditory pitches. Systematic variations in the duration of the first click train (T1) from 4 to 0.5 seconds were implemented for Reg_4__–4__.06_, while maintaining the duration of the second train (T2) at a constant 2 seconds (Fig. 3A). The change response of an example channel exhibited a gradual increase in amplitude with the extension of the T1 duration (*P* < 0.001, ANOVA, Fig. 3B). Specifically, the change response was significantly stronger under the 4-second condition than under the 1-second condition for both Monkey C and Monkey X (*P* < 0.001 for both monkeys, ANOVA with post-hoc test, Fig. 3C). These results accentuated the capacity of temporal merging to integrate auditory stimuli over durations that exceeded 1 second.

Considering the inherent limitations of shortening T1 duration further due to potential overlap between the onset and change responses, an alternative method was employed to determine the minimum duration that was essential for temporal merging into an auditory pitch. Two click trains with ICIs of 4 and 4.06 ms, respectively, were continuously alternated and the duration of these alternating trains was systematically varied from 500 to 30 ms (Fig. S9A). This design aimed to evaluate whether the AC could merge these click trains into pitches, manifesting as oscillatory responses that corresponded to the switching rate of the trains. Clear oscillations were observed at durations of 500, 250 and 125 ms whereas no such oscillations were discerned at 60 and 30 ms in an example site (Fig. S9B). Fast Fourier transform analysis confirmed the presence of corresponding peaks at 500, 250 and 125 ms (Fig. S9C). Notably, a significant peak was identified at 60 ms (*P* < 0.001, *t*-test, corresponding to 16.6 Hz), although no peak emerged at 30 ms (*P* > 0.05, *t*-test, corresponding to 33.3 Hz). Topographical mapping revealed significant oscillations in both monkeys at 500, 250 and 125 ms (Fig. S9D and E, *t*-test conducted at each recording site). However, no significant oscillations were detected at 30 ms in both monkeys (Fig. S9D and E, *t*-test conducted at each recording site). To ascertain that the absence of a peak at 30 ms did not indicate a failure to track such high-frequency changes intrinsically, a similar stimulation paradigm was applied by using two tones (200 and 250 Hz) (Fig. S10A). The results revealed that AC could indeed track changes at a rate of ≥33 Hz (Fig. S10B–E, *P* < 0.001 for both monkeys in Fig. S10B and C). Consequently, the findings suggested that the minimum duration required for temporal merging into auditory pitches lies within the range of 30–60 ms.

### The effect of ICI length

Following the exploration of train duration, we delved into another crucial temporal parameter: the impact of the ICI length on temporal merging. When systematically varying the ICI while maintaining a constant ratio of ICIs between the two click trains (Fig. 3D), we observed notable shifts in the change response. As the ICI increased, the change response at the example site diminished; specifically, Reg_4__–4__.06_ elicited the most substantial response (*P* < 0.05, ANOVA with post-hoc test, Fig. 3E), Reg_8__–8__.12_ displayed a significant response (*P* < 0.001, *t*-test) and no discernible change response was detected in Reg_20__–2__0.3_, Reg_40__–4__0.6_ and Reg_80__–8__1.2_ (*P* > 0.05, *t*-test). Across the population channels, the CRI exhibited a consistent decrease with increasing ICI for both monkeys (*P* < 0.001 for both monkeys, ANOVA, Fig. 3F). In the case of Reg_8__–8__.12_, both monkeys exhibited significant responses (*P* < 0.001, *t*-test for both monkeys) whereas significance was not observed for Reg_20__–2__0.3_ or the longer ICI conditions in the population (*P* > 0.05, *t*-test for both monkeys). However, a few channels demonstrated both a change response and a synchronized response to individual clicks under Reg_20__–2__0.3_ (Fig. S11), suggesting the co-occurrence of individual-click responses and switch-pitch responses. This co-occurrence indicates the presence of two distinct signals: one associated with individual-click synchronization and another with temporal merging.

### The effect of ICI contrast

Next, we examined how the change response was affected by the difference in the ICI, i.e. the ICI contrast. We varied the ICI contrast from 1% to 0.25% in a systematic way (Fig. 3G). As the difference increased, the change responses of an example site became more prominent (*P* < 0.001, ANOVA, Fig. 3H). Across the population channels, an increase in the difference also enhanced the strength of the CRI (*P* < 0.001 for both monkeys, ANOVA, Fig. 3I). Remarkably, even a 0.25% difference (Reg_4__–4__.01_) could elicit significant change responses in the population with the average CRI of 0.03 (*P* < 0.001, *t*-test, Fig. 3I, left panel) and 0.02 (*P* < 0.001, *t*-test, Fig. 3I, right panel) for Monkey C and X, respectively, indicating a high temporal resolution in temporal merging.

### The effect of train regularity

In our examination thus far, attention has been primarily devoted to click trains with consistent ICIs, termed as regular click trains. However, this led us to ponder on the effect of inconsistent ICIs within click trains, which we categorize as irregular click trains. In line with the Reg_4__–4__.06_ construct, we formulated an irregular transitional click train, Irreg_4__–4__.06_, with average ICIs of 4 and 4.06 ms, in which the variance of the ICI was set to half of each respective average ICI (*µ*/2), following a Gaussian distribution (Fig. S12A). Interestingly, while clear responses were observed for Reg_4__–4__.06_ (*P* < 0.001, *t*-test), Irreg_4__–4__.06_ failed to elicit comparable reactions in the exemplary site (*P* = 0.52, *t*-test, Fig. S12B). A comparative analysis of the change response magnitude across various sites showcased a pronounced disparity in response strength between regular and irregular patterns; most data points were observed beneath the diagonal line across both primates (two-way ANOVA, *F*(1, 244) = 296.1, *P* < 0.001, Fig. S12C). A notable distinction between the two stimulation patterns was also evident from the response traces of all recording sites (Fig. S12D–G). Supporting these physiological findings, psychophysical analyses further indicated that deviations in regular patterns were more readily detected compared with those in irregular ones with a variance of *µ*/2 for both monkeys (*P* < 0.001, Wilcoxon rank-sum test for both monkeys, Fig. S8D and E).

To further confirm that the observed change response was a result of temporal integration rather than transient alterations, a control experiment was conducted. In this experiment, the Irreg_4__–4__.06_ stimulus with a variance of *µ*/2 was designed, incorporating two successive intervals of 4 ms followed by two of 4.06 ms around the transitional point (Fig. S13A). This set-up was intended to maintain consistent transient changes between the Irreg_4__–4__.06_ and Reg_4__–4__.06_ patterns. However, despite these controlled conditions, the response elicited by Irreg_4__–4__.06_ was significantly weaker compared with that of Reg_4__–4__.06_—a distinction that was clearly demonstrated by the response patterns of the example recording site (*P* < 0.001, *t*-test, Fig. S13B). Further, a comprehensive comparison of the response strengths across various recording sites reinforced this observed discrepancy (two-way ANOVA, *F*(1, 244) = 306.6, *P* < 0.001 in Fig. S13C), which was also evident from the response traces of all recording sites (Fig. S13D–G).

Quantitative evaluation of this regularity effect entailed experimentation with varied variances for each train, encompassing *µ*/400, *µ*/200, *µ*/100 and *µ*/50, in which *µ* represents the mean ICI (either 4 or 4.06 ms) (Fig. 3J). When the response profile of a representative recording site was scrutinized, there was negligible difference between *µ*/400 and *µ*/200 in the example site (*P* = 0.83, ANOVA with post-hoc test, Fig. 3K). However, with an upsurge in variance, the response started to attenuate noticeably (*P* < 0.001, ANOVA, Fig. 3K). Analogously, on the population scale, as the variance escalated, there was a commensurate decline in the CRI for both monkeys (*P* < 0.001 for both monkeys, ANOVA, Fig. 3L).

The spatial distribution of the CRI across both subjects additionally highlighted the influence of these four factors (train duration, ICI length, ICI contrast and train regularity) on the change response (Fig. S14).

### Origin of the temporal merging

**Figure 4.**
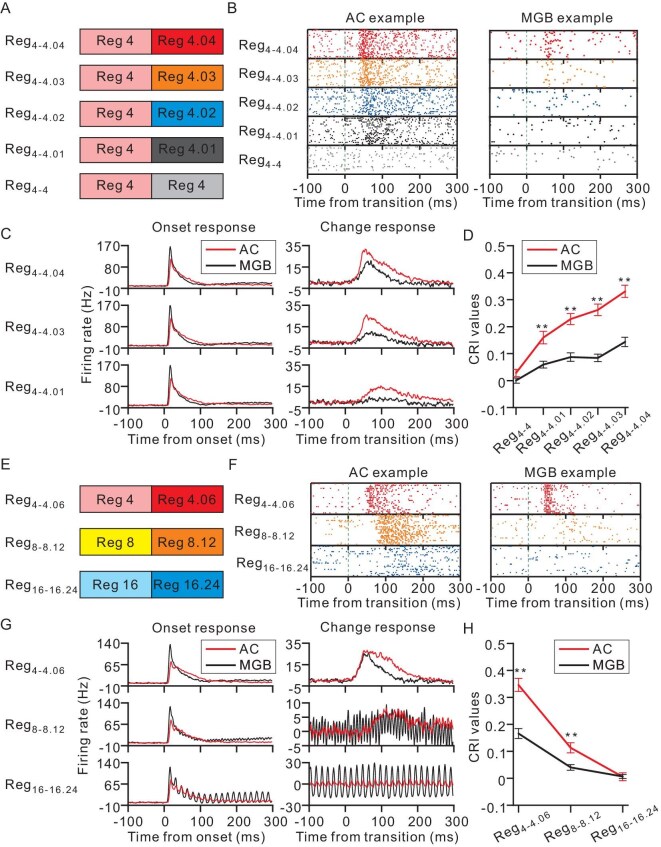
Differential change responses in the AC and the MGB to ICI contrast and ICI length. (A–D) Analysis of ICI contrast. (A) The stimulation set-up employed four levels of ICI contrast for the transitional trains: 4–4.01, 4–4.02, 4–4.03 and 4–4.04 ms, with a control click train at a constant interval of 4 ms. These blocks were presented randomly. (B) Raster plots illustrate neuronal responses to varying ICI contrasts in example neurons from the AC (left panel) and the MGB (right panel), with the dashed line marking the transition point. (C) Population Peri-stimulus-time histograms (PSTHs) subtracted from the respective baselines (a pre-stimulus window ranging from –100 ms to stimulus onset) for both AC and MGB compare the onset (left panel) and change responses (right panel) between AC (*n* = 142) and MGB (*n* = 141) for Reg_4__–4__.04_ (top row), Reg_4__–4__.03_ (middle row) and Reg_4__–4__.01_ (bottom row). (D) Average CRI values plotted against ICI contrast for AC (*n* = 142) and MGB (*n* = 141). **P < 0.001, two-way ANOVA with post-hoc test for each ICI contrast condition. (E–H) Exploration of ICI length. (E) The stimulation set-up involved three transitional click train combinations: 4–4.06, 8–8.12 and 16–16.24 ms. The blocks were presented in a random order. (F) Raster plots demonstrate responses to different ICI lengths in example neurons from AC (left panel) and MGB (right panel). The dashed line indicates the transition point. (G) Population PSTHs subtracted from the respective baselines (a pre-stimulus window ranging from –100 ms to stimulus onset) for both AC and MGB detail the onset (left panel) and change responses (right panel) for AC (*n* = 142) and MGB (*n* = 141), featuring Reg_4__–4__.06_ (top row), Reg_8__–8__.12_ (middle row) and Reg_16__–1__6.24_ (bottom row). (H) CRI values as a function of the ICI length for AC (*n* = 142) and MGB (*n* = 141) are depicted. **P < 0.001, two-way ANOVA with post-hoc test for each ICI length condition.

After the factors that were influencing the temporal merging were characterized, our next objective was to delve into the neuronal mechanisms underlying this phenomenon. We firstly aimed to elucidate the origin of the temporal merging and conducted a comparative analysis of the neuronal responses to the transitional trains between the AC and the medial geniculate body (MGB) while considering two critical factors: ICI contrast (Fig. 4A–D) and ICI length (Fig. 4E–H).

In our investigation of ICI contrast, we maintained the ICI of the initial click train at a constant 4 ms while varying the ICI of the subsequent train. The ICI contrasts were set to 0, 0.25%, 0.5%, 0.75% and 1%, corresponding to ICIs of 4, 4.01, 4.02, 4.03 and 4.04 ms, respectively, for the second train (Fig. 4A). An exemplary neuron from the AC, as shown in the left panel of Fig. 4B, demonstrated a significant change response even at the minimal 0.25% ICI contrast—a response that was not paralleled in the example MGB neuron (right panel of Fig. 4B). Population data analysis (Fig. 4C) revealed no significant change response in the MGB for ICI contrasts ranging from 0.25% to 0.75% (*P* = 0.28 for 0.25%, *P* = 0.1 for 0.5% and *P* = 0.14 for 0.75%, *t*-test), with a noticeable response emerging only at the 1% contrast (*P* < 0.05, *t*-test). In stark contrast, AC neurons displayed significant change responses for as low as a 0.25% ICI contrast (*P* < 0.001, *t*-test), suggesting the occurrence of change responses in the AC even when such responses were absent in the MGB population for subtle ICI contrasts. Notably, under the largest ICI contrast condition (Reg_4__–4__.04_), the onset responses in the AC population tended to have a marginally later latency, while the change responses occurred earlier compared with those in the MGB (Fig. S15). Furthermore, AC neurons exhibited considerably stronger change responses across all four ICI contrast conditions (*P* < 0.001 for all conditions, two-way ANOVA with post-hoc test, Fig. 4D).

Regarding the ICI length, we explored three conditions: Reg_4__–4__.06_, Reg_8__–8__.12_ and Reg_16__–1__6.24_ (Fig. 4E). The robust response of an example AC neuron to the Reg_16__–1__6.24_ condition is depicted but not that of the example MGB neuron in Fig. 4F. At the population level, robust change responses were detected for both the MGB and the AC under the Reg_4__–4__.06_ condition (for topographical distribution, see Fig. S16C and E). However, a significant response was evident in the AC but not in the MGB for the Reg_8__–8__.12_ condition (*P* < 0.05 for AC and *P* = 0.29 for MGB, *t*-test, Fig. 4G), suggesting the existence of change responses in AC even when none was observed in the MGB population for longer ICI lengths. Neither the MGB nor the AC exhibited significant responses in the Reg_16__–1__6.24_ condition in the neuronal population (*P* > 0.05, *t*-test, Fig. 4G). Notably, AC neurons showed considerably stronger change responses under both the Reg_4__–4__.06_ and Reg_8__–8__.12_ conditions (*P* < 0.001 for both conditions, two-way ANOVA with post-hoc test, Fig. 4H).

To emphasize the distinction between the AC and the MGB, we can compare the percentage of neurons that showed significant change responses. For ICI contrast, 47.9% of the AC neurons exhibited significant change responses whereas only 21.3% of the MGB neurons showed significant responses under the largest ICI contrast condition (1%). Furthermore, 23.2% of the AC neurons displayed significant change responses compared with only 2.8% of the MGB neurons under the minimum ICI contrast condition (0.25%). For ICI length, 57.8% of the AC neurons and 34.8% of the MGB neurons exhibited significant change responses under Reg_4__–4__.06_ whereas, under Reg_8__–8__.12_, 13.4% of the AC neurons and 6.4% of the MGB neurons displayed significant change responses. Interestingly, only 4 out of 142 AC neurons showed significant responses in Reg_16__–1__6.24_ while none of the 141 MGB neurons exhibited significant change responses. The percentage of neurons that exhibited significant response changes differs significantly between the AC and the MGB across all conditions (*P* < 0.05, chi-square test; Table S1).

### Clinical implication for temporal merging

**Figure 5.**
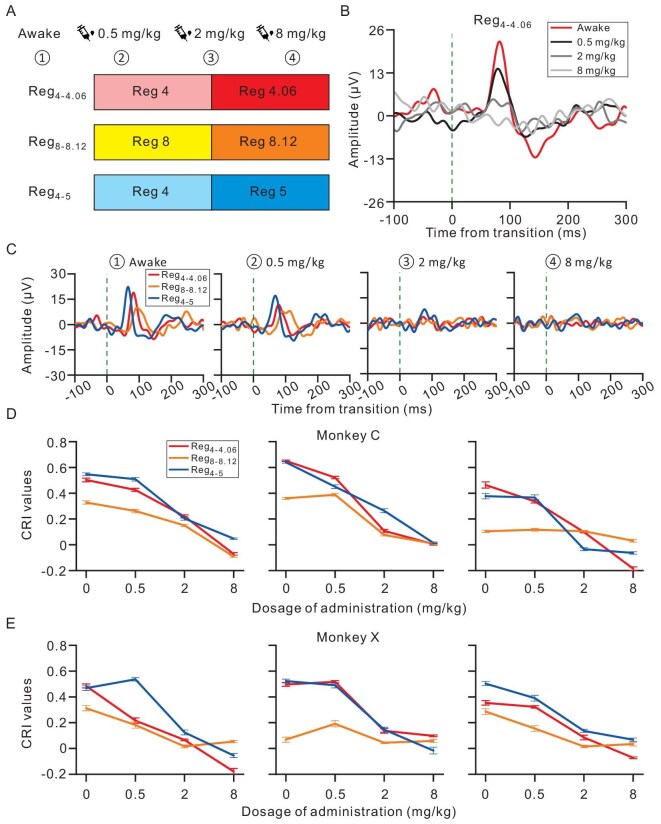
Change response acted as a biomarker of anesthesia depth. (A) Experimental design. Three combinations of click interval were chosen in the transitional trains: 4–4.06, 8–8.12 and 4–5 ms. Anesthesia sessions were conducted sequentially with escalating ketamine doses: awake, 0.5, 2 and 8 mg/kg. The three stimulation blocks were presented randomly in each session. (B) ECoG response of a representative site to Reg_4__–4__.06_ under varying anesthesia levels. (C) Comparative responses to Reg_4__–4__.06_, Reg_8__–8__.12_ and Reg_4__–5_ throughout the four anesthesia stages. (D and E) CRI values as a function of the administration dosage across three experimental sessions for monkey C (D) and monkey X (E), with each subplot representing a distinct session.

Our study has elucidated four primary determinants that modulate the change response: train duration, ICI length, ICI contrast and train regularity. These determinants provide invaluable metrics for dissecting the nuances of brain functionality associated with temporal integration. Thus, the change response emerges as a promising biomarker for neuropsychiatric conditions. Venturing further into this potential clinical application, we assessed the influence of the anesthetic agent ketamine on the change response. Continuous recordings were conducted across four distinct conditions sequentially: awake, followed by incremental doses of ketamine at 0.5, 2 and 8 mg/kg (Fig. 5A). Notably, there was a systematic attenuation of the change response to Reg_4__–4__.06_ in alignment with these conditions at a representative ECoG recording site (Fig. 5B). Additionally, the change response to the three evaluated transitional click trains (Reg_4__–5_, Reg_4__–4__.06_, Reg_8__–8__.12_) in the representative site consistently diminished from the awake state to the deepest anesthetized condition (two-way ANOVA, *F*(3, 360) = 483, *P* < 0.001, Fig. 5C). This consistent trend was corroborated across three sessions on different days for both monkeys (three-way ANOVA for both monkeys, *F*(3, 3168) = 3528, *P* < 0.001, Fig. 5D and E), underscoring a stable impact of ketamine on the change response.

## DISCUSSION

When the ICI is short, the AC is unable to synchronize with each pulse within the train. Instead, it responds only to the first few clicks and then ceases to respond to subsequent clicks due to adaptation (Fig. S2). However, the introduction of the transitional train leads to a robust change response in the previously adapted AC (Fig. 1). Notably, this change response cannot be attributed to alterations in the frequency that correspond to the click-repetition rate in the train (Fig. S5), the transient change in the click interval (Fig. S6) or energy imbalance (Fig. S7), but rather requires temporal integration (Fig. 2). Subsequent psychophysical experiments further support that click trains with different ICIs were perceived as distinct pitches (Fig. S8). Consequently, the change response appears to be related to the perceptual shift between auditory pitches formed through temporal merging. Furthermore, our findings reveal that this signal is significantly influenced by four key factors: the duration of the train (Fig. 3A–C, Figs S9 and S10), the ICI length (Fig. 3D–F), the ICI contrast (Fig. 3G–I) and the regularity of the train (Fig. 3J–L and Fig. S12). Additionally, temporal merging varies with the brain state across different levels of anesthesia (Fig. 5). These results suggest the potential utility of these factors as biomarkers for psychiatric diseases. To probe the origin of temporal merging, a comparison between the AC and the MGB was conducted. The results support the notion that temporal merging originates in the cortex (Fig. 4).

### Change response in the transitional click train as a marker of temporal integration

Auditory research utilizing click trains as stimuli has unveiled intricate neuronal responses in the auditory system. Neurons display a remarkable capability for precise temporal coding where individual spike activities precisely align with specific intervals between the clicks [17]. Despite the prominence of this temporal alignment, rate coding emerges as another vital mechanism. Particularly at accelerated click rates [18], Lu *et al.* [10] identified two distinct populations of neurons: one that synchronizes to slow sound sequences and another that encodes rapid events through firing rates. However, these studies have predominantly focused on understanding how individual clicks within the train are represented, paying limited attention to the holistic representation of the click train as a unified auditory event. It is worth noting that, psychophysically, when the ICI is less than ∼33 ms, click trains are often perceived as pitch [14]. This presents a considerable challenge: to disentangle the neural responses that are elicited by individual clicks from those triggered by the perception of the click train as pitch. However, neither the neuronal nor the ECoG response could adequately represent the auditory event through temporal integration with click trains in the previous research [10,17,18]. To navigate this intricacy, we put forth the concept of a transitional train, as illustrated in Fig. 1. Although transitional trains have been used to probe pitch sensitivity, the nature of the change response that is elicited by transitional trains remains unclear [15,16]. Within a transitional train, the neuronal response to the individual clicks may undergo adaptation during the presentation of the first train and, in some cases, it may even cease to respond. However, the introduction of the second train not only sustains the presentation of individual-click stimuli, but also initiates a perceptual switch, signifying a transition between distinct auditory pitches. Thus, when the ICI is small (e.g. ∼4 ms), only the onset response is detected and subsequent clicks in the train do not elicit further responses, suggesting strong adaptation (Fig. S2B and C). Simultaneously, listeners perceive click trains with such ICIs as a unified auditory event (Figs S1 and S8) and, consequently, the introduction of a transitional train triggers a change response in the adapted cortex, followed by subsequent adaptation (Fig. 1B). This change response is not solely attributed to local temporal changes (Fig. S6), but is linked to temporal integration (Fig. 2). Therefore, the transitional train offers a convenient approach to investigate temporal integration. This innovative approach allows us to disentangle the neural representation of individual clicks from that of the holistic auditory event, shedding light on the intricate process of temporal integration in auditory perception.

Auditory research has historically focused on the frequency domain, influenced by the tonotopic organization of the auditory system in which distinct frequencies are processed separately along the auditory pathway [1]. However, the crucial role of the temporal dimension in auditory processing cannot be overlooked. Aspects such as rhythm, timing and complex sound pattern recognition are deeply rooted in temporal information [2]. This temporal dimension underpins essential aspects of speech and music perception, as well as the differentiation of environmental sounds [3]. Contemporary neuroimaging and electrophysiological methods have deepened our understanding of temporal integration mechanisms in oral language, showcasing a hierarchical temporal integration structure in the human brain [19,20]. However, a knowledge gap exists in temporal integration in non-human animals. The main hindrance has been the absence of a neuronal signature for temporal integration, stalling neuronal-level research and animal studies. The change response to the transitional train in our study offers a promising avenue through which to explore this intricate area.

In addition to signal for temporal integration, our study elucidates the neuronal mechanisms underlying pitch perception evoked by click trains. Our findings highlight the role of temporal integration within the AC as a key process in pitch perception. Traditional theories differentiate between resolved and unresolved harmonics based on the ability of the auditory system to segregate individual harmonic components [21,22]. Resolved harmonics arise from distinct components that are processed by separate auditory filters whereas unresolved harmonics involve closely spaced components within a single filter, relying on temporal coding for pitch extraction. Interestingly, sounds with the same repetition rate but very different spectra often have the same pitch, while sounds with similar spectra can produce significantly different pitches [21,22]. This demonstrates that the frequency-to-place mapping performed by the cochlea does not necessarily correspond to a frequency-to-pitch mapping [23]. The temporal pitch induced by click trains is distinct because it relies solely on the temporal regularity of successive auditory events rather than the spectral components [16,23–25]. Our study provides compelling neuronal evidence that supports this process, demonstrating that the change response of the AC encapsulates the integration of temporal information into a unified auditory pitch (Fig. 2). Previous researchers used transitional click trains to investigate temporal pitch sensitivity [15] and observed the change response in electroencephalography (EEG) signals of cats [16]. Our insertion experiments further explored the nature of the change response, focusing on temporal integration (Fig. 2). Additionally, we examined the change response at the neuronal level and compared responses between the AC and the MGB (Fig. 4). These findings position the AC as the central locus for temporal pitch perception in click trains. However, it is important to note that pitch is a perceptual variable rather than a physical quantity or a direct neuronal representation. Therefore, future work should employ behaving monkeys, from the perspective of perception, to address the relationship between the change response and pitch-switch detection, as well as explore the duration of click trains required for integration. This will help to clarify the nature of pitch perception and temporal integration in auditory processing.

### Neuronal mechanism underlying change response in the transitional click train

Change responses present notable disparities compared with pure tones and onset responses. Firstly, click trains exhibit enhanced resolution, as demonstrated by the significantly stronger response to Reg_4__–4__.06_ compared with the corresponding frequency change of the tone (Tone_250__–2__46_, Fig. S5). Remarkably, even a minute difference of 0.25% (Reg_4__–4__.01_) in the ICI can elicit a robust response, indicating heightened sensitivity (Fig. 3G–I and Fig. 4C and D). Psychophysically, regular click trains also demonstrate increased discernment relative to pure tones (Fig. S8D and E). Secondly, the latency of the change response is considerably longer than that of the onset response (Fig. S4), implying a distinct origin for the change response. Thirdly, the change response to the transitional train cannot keep pace with rapid rates of switch, such as 33.3 Hz, which can be tracked by responses to pure tones (Fig. S10). In summary, the distinctive properties of the change response, when contrasted with pure tones and onset responses, suggest a divergent underlying origin. While the onset response and response to pure tones arise from the peripheral system (e.g. base membrane), temporal merging may represent a novel process that involves more complex computations in the central nervous system, rather than relying solely on frequency resolution from the base membrane.

Direct evidence in support of this notion is derived from the comparison between the AC and the MGB (Fig. 4 and Table S1). In the response to Reg_4__–4__.01_ (Fig. 4C and D), AC neurons exhibit a significant change response (33/142) while MGB neurons rarely respond (4/141). Similarly, in the response to Reg_8__–8__.12_ (Fig. 4G and H), AC neurons also display a significant change response (19/142) whereas MGB neurons rarely respond (9/141). Thus, in contrast to the onset response, the change response may exist in the AC under conditions where the MGB shows no change response in the neuronal population. Moreover, under other conditions tested (Fig. 4), AC neurons exhibited much stronger responses than MGB neurons, even though the onset response was similar to those of MGB neurons. It is important to mention that the majority of AC neurons that we recorded likely belonged to the primary auditory cortex whereas the MGB neurons were from all main divisions, encompassing ventral, dorsal and medial parts (Fig. S16). Taken together, we posit that the change response in the AC has a cortical origin rather than originating solely from thalamic input. Considering the extensive interconnections between the sensory cortex and the thalamus [26,27] and the earlier occurrence of the change response in the AC compared with the MGB (Fig. 4C, D, G and H), it is plausible that the change response in the MGB arises from corticothalamic feedback. The neuronal circuitry underlying the cortical and thalamic change responses needs further investigation in the future.

### Change response in the transitional click train as a biomarker in clinical applications

Four key factors influence the change response and are consequently related to temporal merging, offering diverse metrics for characterizing temporal integration and potentially serving as valuable tools in clinical applications.

The first factor is the duration of the click train. The minimal duration required for a click train to merge into an auditory pitch is ∼60 ms for a 4-ms ICI (Fig. S9). In human studies, listeners can reliably identify sound categories with just 25 ms of duration [28], which aligns with the results of the tracking tone changes in the monkeys (Fig. S10). Interestingly, the magnitude of the change response continues to increase even with click train durations of >2 seconds (Fig. 3A–C), suggesting prolonged temporal integration during temporal merging. The second factor is the length of the ICI. Our research found that, when the ICI is >20 ms, the auditory system fails to merge the clicks into a single auditory pitch, as evidenced by the absence of a change response except following each pulse stimulation (Fig. 3D–F). This result is consistent with psychophysical findings in which humans can detect gaps and fail to perceive the click train as a unified sound when the ICI is >29.6 ms (Fig. S1). The third factor is the difference between the ICIs (Fig. 3G–I). The superior resolution of ICIs relative to pure tones suggests the involvement of a dedicated neuronal circuit with very fine temporal resolution in temporal merging. The fourth factor is the regularity of the click train, which not only characterizes the temporal structure, but also requires extended time for integration to extract the regularity of the train, reflecting context-dependent temporal integration (Fig. 3J–L).

These factors hold promise as potential biomarkers for mental disorders. To further explore this possibility, we manipulated the state of the brain by using the anesthetic drug ketamine, controlling the anesthetic level by varying the dosage of the drug (Fig. 5). The change response systematically decreased with increasing drug dosage and this relationship remained consistent over three recording sessions on different days. These results suggest a direct relationship between the change response and the anesthetic level, indicating the potential use of the change response as a marker for monitoring anesthetic levels. The importance of effective anesthesia monitoring during surgical procedures is paramount, as it ensures patient safety and optimal surgery outcomes. However, the current standard practice of using EEG for monitoring anesthesia levels faces challenges in terms of accuracy and reliability [29]. The observed change response to the transitional train provides an innovative pathway for refining anesthesia-monitoring techniques. Further extending the clinical applicability of our research, we propose the use of the transitional trains for the assessment of psychiatric conditions. Temporal integration, which is a central component of brain functionality [30,31], has been found to be compromised in conditions such as schizophrenia [32], autism spectrum disorders [33,34], attention deficit hyperactivity disorder [35] and Parkinson's disease [36]. Given these findings, the signal of temporal integration might be poised to emerge as a pivotal biomarker for broader clinical diagnostics.

## MATERIALS AND METHODS

The experimental protocols strictly conformed to the guidelines approved by the State Council of China (GB 14925-2010) and were sanctioned by the Bioethics Committee of Zhejiang University (ZJU20200148). Detailed materials and methods are available in the Supplementary files.

## Supplementary Material

nwaf026_Supplemental_Files
