# Supplementary material for: Temporal merging into pitch with click train in the macaque auditory cortex
Source: Natl Sci Rev. 2025 Jan 22;12(6):nwaf026. doi: 10.1093/nsr/nwaf026 (PMC12139000; doi:10.1093/nsr/nwaf026)
Supplement: nwaf026_Supplemental_Files [file nwaf026_supplemental_files.zip › Supplementary Figure Legends.docx]

**Figs. S1 to S****17**

**Fig. S1 Gap detection task in click train sequences**

(**A**) This schematic outlines the gap detection task. Participants were positioned in a chair facing a keyboard and speaker. The speaker emitted click trains of 1024 ms duration with ICIs varying from 4 ms to 256 ms. Following each click train, a cue was presented after 800 ms. Participants were tasked to press the right key within 700 ms after the cue if a gap was discernible in the click train. Alternatively, if the click train was perceived as a continuous auditory event, the left key was to be pressed.

(**B**) This plot aggregates the responses from all participants (N = 22). The horizontal axis shows the ICIs of the click trains, while the vertical axis represents the frequency of left key presses, indicative of continuous sound perception. Individual participant responses are depicted in pink, with the mean pressing ratio illustrated by the red line. Error bars denote the standard error (SE) of the mean.

(**C**) This plot displays the distribution of the psychological thresholds for gap detection among the participants. The arrow indicates the mean threshold value across the participants.

**Fig. S2 ECoG response to click train.**

(**A**) Illustration of the position of the ECoG array above the AC of a monkey.

(**B**) The responses of one example site to click trains with different ICIs: 512 ms (top row), 32 ms (middle row), and 4 ms (bottom row).

(**C**) FFT of the response to click train with ICI of 32 ms (left panel) and 4 ms (right panel). The arrows point to the frequencies that match the click repetition rates.

**Fig. S3 Change response in ICI-reversed transitional train.**

(**A**) The structure of the transitional train. Each block consists of 5-second regular click train with 4.06-ms ICI followed by another 5-second regular click train with 4-ms ICI. The transitional train was labeled as Reg_4.06-4_. The black pulse represents the common pulse shared by both trains and the green dashed line marks the transition point.

(**B**) The responses of one example channel to Reg_4.06-4_. The green dashed line shows the transition point time.

(**C**) A magnified view from (B) showing the window from -100 to 600 ms relative to the transition point in the transitional train.

(**D-E**) ECoG recording reveals the responses to Reg_4.06-4_ for all 64 channels in two monkeys. Each subplot corresponds to one recording site with a window from 0 to 600 ms relative to the transition point. The black box indicates the location of the example channel in (B).

**Fig. S4 Comparison of change response and onset response to Reg_4-4.06_.**

(**A**) The example channel showing the change response (red) and onset response (blue). The arrows point to first peaks of the traces.

(**B**) Scatter plot comparing latency for change response and onset response for both monkeys. Each dot represents an individual ECoG recording site; black circles denote data from Monkey C, while gray triangles correspond to Monkey X.

(**C-D**) Histograms showing the latency of change response (red) and onset response (blue) for Monkey C (C) and Monkey X (D), respectively, with arrows and accompanying numbers marking the mean latencies.

**Fig. S5 Comparison of AC responses to frequency change with pure tones and ICI change with click trains.**

(**A**) Stimulation setting. A 250 Hz tone was presented for 5 s followed by another 246 Hz tone for 5 s (top row). This sound was labeled as Tone_250-246_. The bottom row shows Reg_4-4.06_.

(**B**) The normalized responses of one example channel to Tone_250-246_ (blue) and Reg_4-4.06_ (red).

(**C**) Scatter plot comparing the normalized CRI values for Tone_250-246_ and Reg_4-4.06_ for both monkeys. Black circles represent Monkey C; gray triangles represent Monkey X.

(**D-G**) ECoG recordings showing the normalized responses to Tone_250-246_ (blue) and Reg_4-4.06_ (red) for all 64 channels in Monkey C (left column) and Monkey X (right column). Each subplot corresponds to one recording site with a time window from -500 to 7000 ms relative to the train onset. The dashed lines in each subplot indicate the start point and the transition point of the sound, respectively. The black boxes in (D) and (F) indicate the location of the example channel in (B).

**Fig. S6 AC responses to transient changes in ICI**.

(**A**) Stimulation setting for inducing a transient change in click train. A single interval of 4.06 ms (highlighted in yellow) was inserted at the 2-s mark into a 3-s click train with a constant ICI of 4 ms (top row). This click train was labeled as Reg_4-4.06-4_. The bottom row shows Reg_4-4.06_, which consists of a 2-s click train with an ICI of 4 ms followed by a 1-s click train with an ICI of 4.06 ms.

(**B**) The responses of one example channel to Reg_4-4.06-4_ (blue) and Reg_4-4.06_ (red).

(**C**) Scatter plot comparing the CRI values for Reg_4-4.06-4_ and Reg_4-4.06_ for both monkeys. Black circles represent Monkey C; gray triangles represent Monkey X.

(**D-G**) ECoG recordings showing the responses to Reg_4-4.06-4_ (blue) and Reg_4-4.06_ (red) for all 64 channels in Monkey C (left column) and Monkey X (right column), respectively. Each subplot corresponds to one recording site with a time window from 0 to 600 ms relative to the transition point. The black boxes in (D) and (F) indicate the location of the example channel in (B).

**Fig. S7 AC responses to transitional train with energy normalization.**

**(A**) Stimulation setup. The transitional train consists of a 2-s click train with an ICI of 4 ms followed by a 1-s click train with an ICI of 4.06 ms. The amplitude of two click trains in the transitional train was normalized according to the square root of the ratio of ICI between two trains, so that the sound energy was constant across time.

(**B**) Responses of one example channel to normalized transitional click train. The dashed line marks the transition point.

(**C**) Enlarged view from (B) showing the window from -100 to 600 ms relative to the transition point.

(**D-E**) ECoG recording reveals the responses to the normalized Reg_4-4.06_ for all 64 channels in two monkeys. Each subplot corresponds to one recording site with a window from 0 to 600 ms relative to the transition point. The black box indicates the location of the example channel in (B).

**Fig. S8 The psychological results of novelty detection in the oddball paradigm**.

(**A**) Schematic diagram of novelty detection behavior. A button is placed in front of the monkey, and a speaker is placed contralateral to the recording side with the same height as the ear of the monkey. Two kinds of blocks of stimuli are presented; in one kind, each block contains several repeated pure tones (or click train) as the standard stimulus and a rare pure tone (or click train) as the deviant stimulus; in the other kind, the block consists of only the same standard stimulus. If the deviant stimulus is presented, the monkey needs to press the button within 600 ms relative to the onset of the deviant presentation to obtain a reward of water; if no deviant sound is presented (control condition), the monkey will get a reward if it doesn’t press the button within 600 ms after the last standard tone in the block.

(**B**) Oddball Stimulation. Four pairs of oddball paradigms were randomly presented. Each pair consisted of one deviant block and one control block, both blocks consisting of 7-10 standard sounds and the last sound could be either the same standard sound (control block) or a different sound (deviant block). One pair utilizes 200 ms regular click trains with 4 ms ICI (standard) and 4.08 ms ICI (deviant); another pair employs irregular click trains averaging 4 ms ICI (standard) and 4.08 ms ICI (deviant); the remaining pairs use pure tones of 250 Hz (standard) with 245 Hz or 200 Hz (deviant). Monkeys must respond promptly to novel sounds for a water reward, while in the control condition, rewards are given if no button press occurs during the decision window.

(**C**) Hit rate in an example session. **: p<0.001, Chi-square test.

(**D-E**) Population data across different recording sessions for Monkey C (D) and Monkey X (E), respectively. Each circle represents one session. Error bars denote the standard error (SE) of the mean.

**Fig. S9 Minimum duration for temporal merging.**

(**A**) Stimulation setup. Two regular click trains with different ICIs (4 and 4.06 ms, corresponding to pink and red colors respectively) were alternately and continuously presented for 10 s. The durations of each click train in each block were randomly selected from these values: 500, 250, 125, 60, 30 ms, corresponding to switching rates of 2, 4, 8, 16.7, and 33.3 Hz, respectively.

(**B**) Responses of one example channel to the alternating click trains with different durations: 500, 250, 125, 60, 30 ms.

(**C**) FFT of the responses to the alternating click trains (red) and FFT of the control condition (black), where only click trains with an ICI of 4 ms were presented for 10 s. The arrows highlight the frequencies corresponding to the switching rates.

(**D-E**) The tonotopic distribution of p values from t-tests comparing the power within the corresponding frequency bands (a range of 0.9 to 1.1 times the switching rate) between the alternating click trains and the control condition for Monkey C (D) and Monkey X (E).

**Fig. S10 Responses to the alternating tones.**

(**A**) Stimulation setup. The alternating tones (200 Hz and 250 Hz) with a duration of 30 ms for each tone (the top row) and alternating click trains (4-ms and 4.06-ms ICI) with a duration of 30 ms for each train (the bottom row) were randomly presented for 10 s.

(**B-C**) Example sites showing the comparison of FFT of the responses to the alternating tones (red) and alternating click trains (black) from Monkey C (B) and Monkey X (C). The arrows highlight the frequencies corresponding to the alternating rates.

(**D-E**) ECoG recordings showing FFT of alternating tones (red) and alternating click trains (black) for all 64 channels in Monkey C (D) and Monkey X (E). Each subplot corresponds to one recording site with FFT analysis. The black boxes indicate the location of example channels in (B) and (C), respectively.

**Fig. S11** **Temporal integration during synchronization.**

**(A)** Responses of one example channel to Reg_20-20.3_ as shown in Fig. 3D. The green dashed line refers to the transition point.

(**B**) A magnified view from (A) showing the window from -100 to 600 ms relative to the transition point in the transitional train. The green dashed line shows the transition point time. The black dotted lines indicate the onset of each click.

**Fig. S12 The effect of train regularity.**

(**A**) Stimulation setup. Each block consists of a 5-second irregular click train with random ICIs averaging 4 ms, followed by another 5-second irregular click train with random ICIs averaging 4.06 ms. The transitional train was labeled as Irreg_4-4.06_ (Top row). The black pulse represents the common pulse shared by both trains and the green dashed line marks the transition point. The bottom row shows Reg_4-4.06_. The two blocks were randomly presented.

(**B**) The responses of one example channel to Irreg_4-4.06_ (blue) and Reg_4-4.06_ (red).

(**C**) Scatter plot comparing the CRI values for Irreg_4-4.06_ and Reg_4-4.06_ for both monkeys. Black circles represent Monkey C; gray triangles represent Monkey X.

(**D-G**) ECoG recordings showing the responses to Irreg_4-4.06_ (blue) and Reg_4-4.06_ (red) for all 64 channels in Monkey C (left column) and Monkey X (right column). Each subplot corresponds to one recording site with a time window from 0 to 600 ms relative to the transition point. The black boxes in (D) and (F) indicate the location of the example channel in (B).

**Fig. S13 AC responses to irregular transitional train**.

(**A**) The top row shows Irreg_4-4.06_ and the bottom row shows Reg_4-4.06_. In the Irreg_4-4.06_, two 4-ms intervals and two 4.06-ms intervals were inserted around the transition point indicated by the yellow background so that Irreg_4-4.06_ has a similar transient change to Reg_4-4.06_ in the stimulation.

(**B**) The responses of one example channel to Irreg_4-4.06_ (blue) and Reg_4-4.06_ (red).

(**C**) Scatter plot comparing the CRI values for Irreg_4-4.06_ and Reg_4-4.06_ for both monkeys. Black circles represent Monkey C; gray triangles represent Monkey X.

(**D-G**) ECoG recordings showing the responses to Irreg_4-4.06_ (blue) and Reg_4-4.06_ (red) for all 64 channels in Monkey C (left column) and Monkey X (right column). Each subplot corresponds to one recording site with a time window from 0 to 600 ms relative to the transition point. The black boxes in (D) and (F) indicate the location of the example channel in (B).

**Fig. S14 The tonotopic distribution of change response for the four factors.**

(**A**) The tonotopic distribution of change response to preceding duration ranging from 0.5 s to 4 s for Monkey C (left) and Monkey X (right).

(**B**) The tonotopic distribution of CRI values for five regular interval combinations: 4-4.06; 8-8.12; 20-20.3; 40-40.6; 80-81.2 ms. Left panel: Monkey C. Right panel: Monkey X.

(**C**) The tonotopic distribution of CRI values for four ICI contrast combinations: 4-4.01; 4-4.02; 4-4.03; 4-4.04 ms. Left panel: Monkey C. Right panel: Monkey X.

(**D**) The tonotopic distribution of CRI values in response to the irregular click train Irreg_4-4.06_, for four levels of ICI variance: µ/400, µ/200, µ/100, µ/50. Left panel: Monkey C. Right panel: Monkey X.

**Fig. S15 Comparison of onset and change latency between AC and MGB.**

(**A**) Population PSTHs subtracted from the baseline (a pre-stimulus window ranging from -100 ms to stimulus onset) comparing the onset responses to Reg_4-4.04_ for AC (red, n=68) and MGB (black, n=30) neurons with significant change responses.

(**B**) Population PSTHs subtracted from the baseline (a pre-stimulus window ranging from -100 ms to stimulus onset) comparing the change responses of Reg_4-4.04_ for the same neuronal populations in (A).

**Fig. S16 Topographical distributions in the AC and MGB.**

(**A**) Three coronal brain MRI sections with highlighted regions (black boxes) in the AC of two macaques, where neuronal recordings were conducted. Each bar represents a schematic penetration for neuronal recording, colored according to the corresponding CF.

(**B**) CF distribution and partitioning of the AC, delineating specific areas: RM (rostral medial area), MM (medio-medial area), CM (caudal medial area), R (rostral area), A1 (primary auditory area), ML (mediolateral area), and CL (caudal lateral area).

(**C**) The distribution of the change response to Reg_4-4.06_ in the AC, with the partition based on the CF distribution in (B). The change response quantifies the difference in neuronal firing rates before and after the transitional point within a 200 ms window.

(**D**) Coronal brain MRI sections with blue boxes indicating the regions of the MGB in two macaques where neuronal recordings were performed. Each bar signifies a penetration for neuronal recording.

(**E**) The distribution of the change response to Reg_4-4.06_ within the MGB for both monkeys.

**Fig. S17 Anatomical location details of ECoG array and reference implantation.**

**(A)** The exposed brain surface, including the lateral sulcus (ls) and superior temporal sulcus (sts), following the opening of the skull and dura mater.

**(B)** The placement of the ECoG electrodes on the brain surface.

**(C)** The location of the reference line (blue line) and the reference electrode (marked by black circles).
