# Supplementary material for: Temporal merging into pitch with click train in the macaque auditory cortex
Source: Natl Sci Rev. 2025 Jan 22;12(6):nwaf026. doi: 10.1093/nsr/nwaf026 (PMC12139000; doi:10.1093/nsr/nwaf026)

Supplementary Figure 1

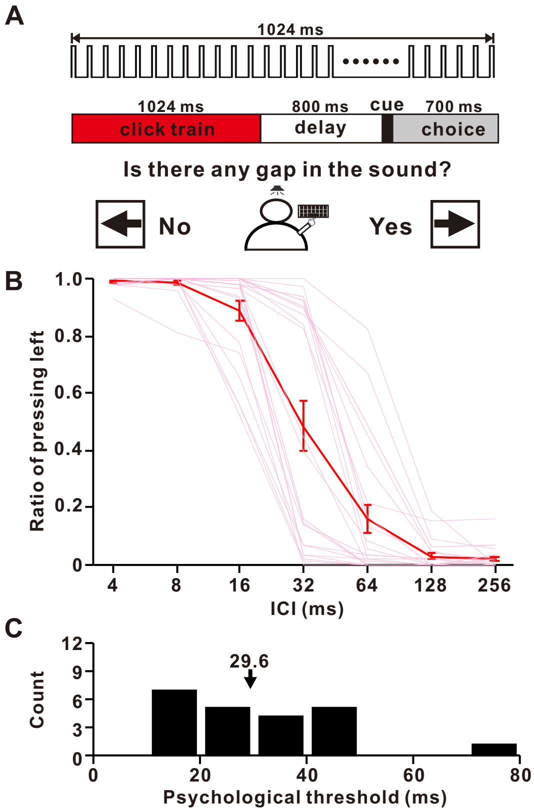

Supplementary Figure 2

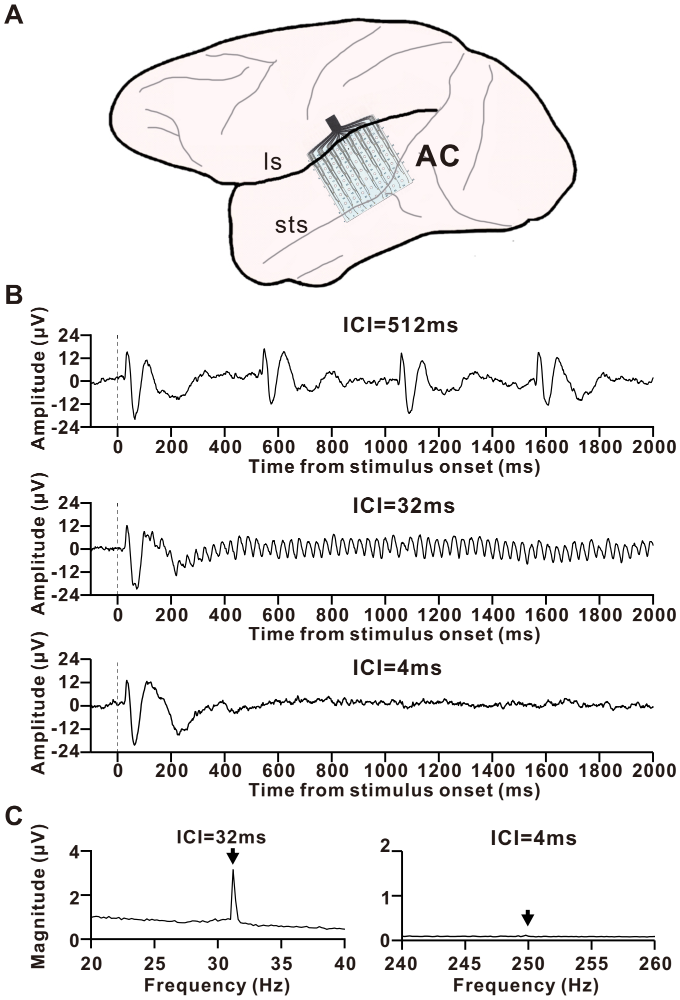

Supplementary Figure 3

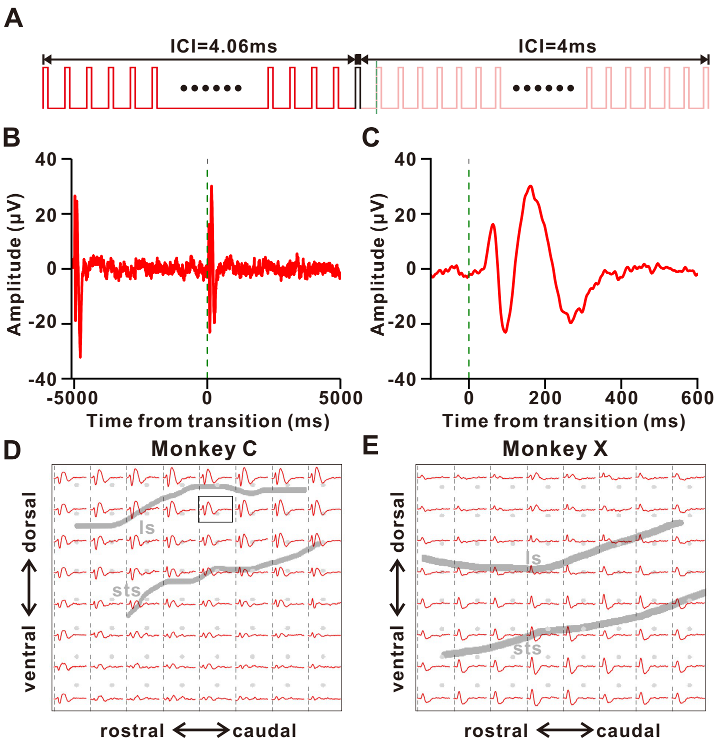

Supplementary Figure 4

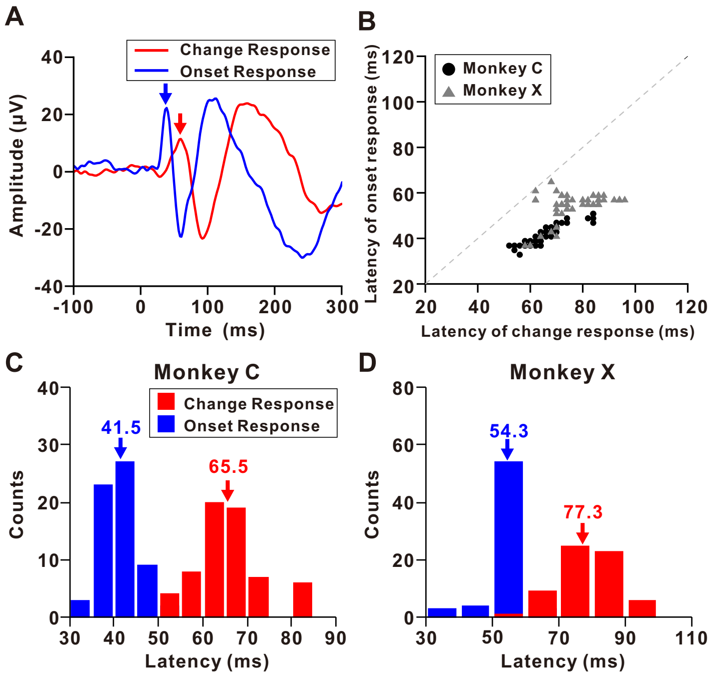

Supplementary Figure 5

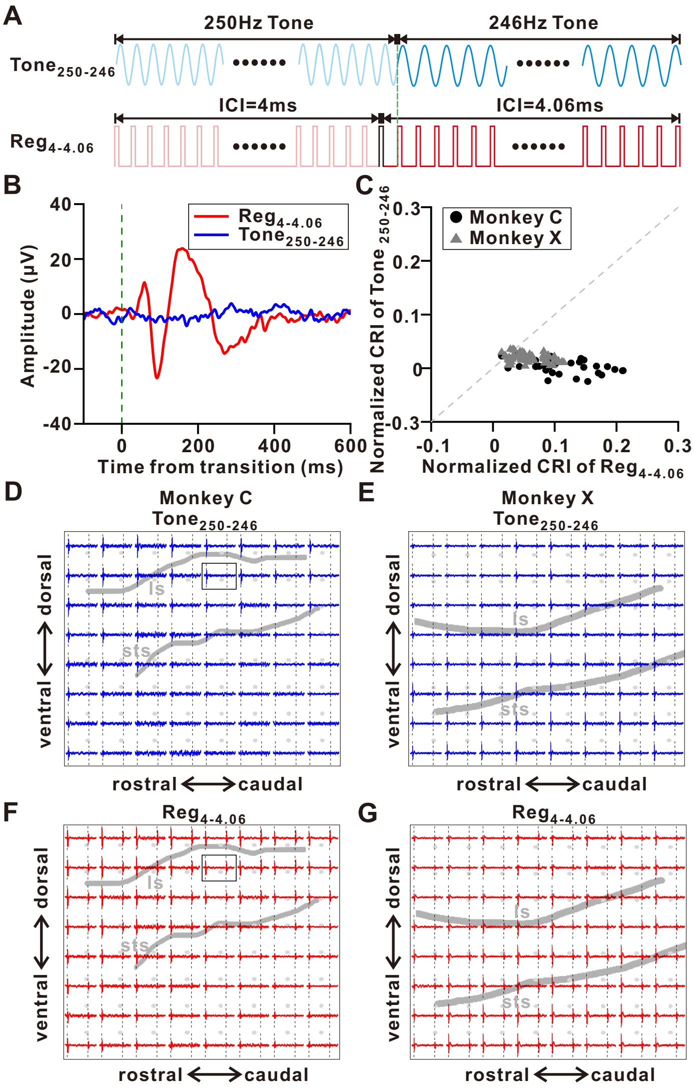

Supplementary Figure 6

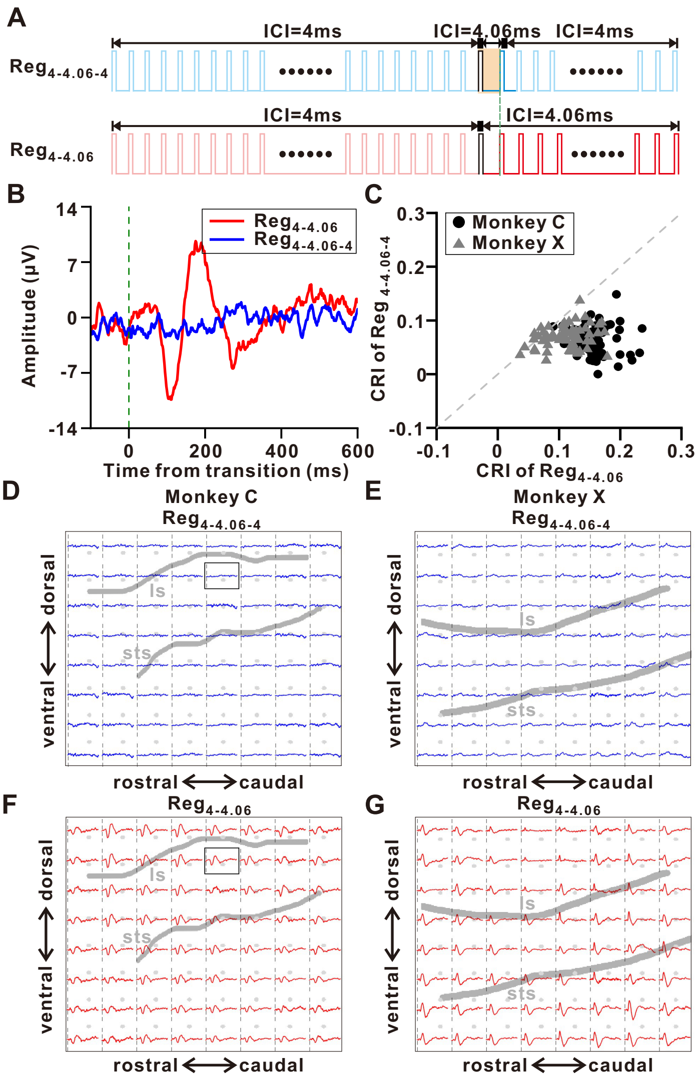

Supplementary Figure 7

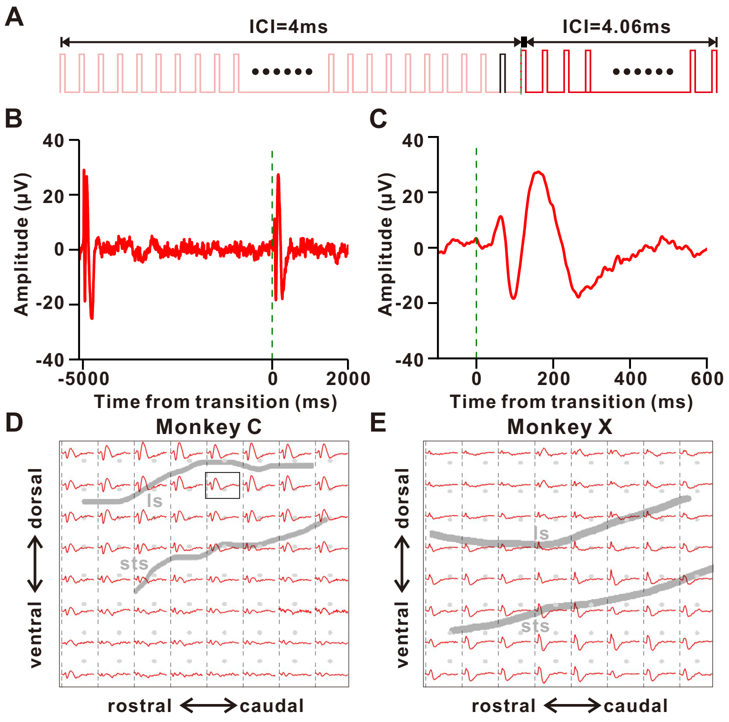

Supplementary Figure 8

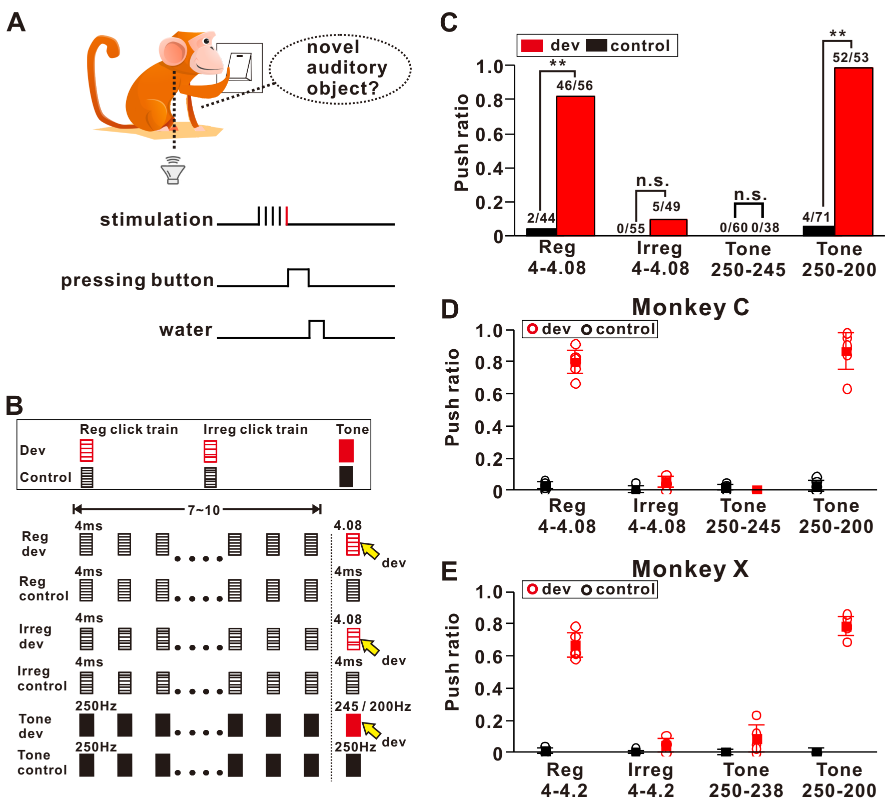

Supplementary Figure 9

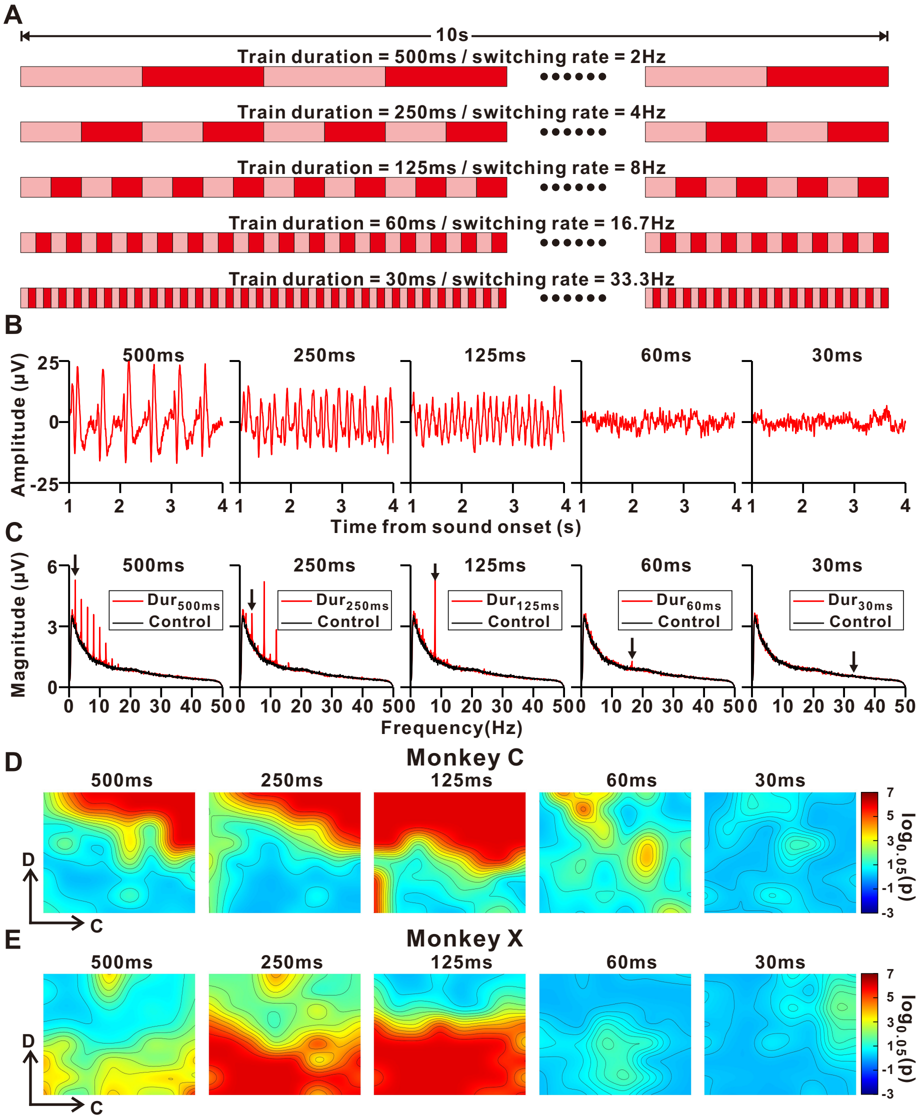

Supplementary Figure 10

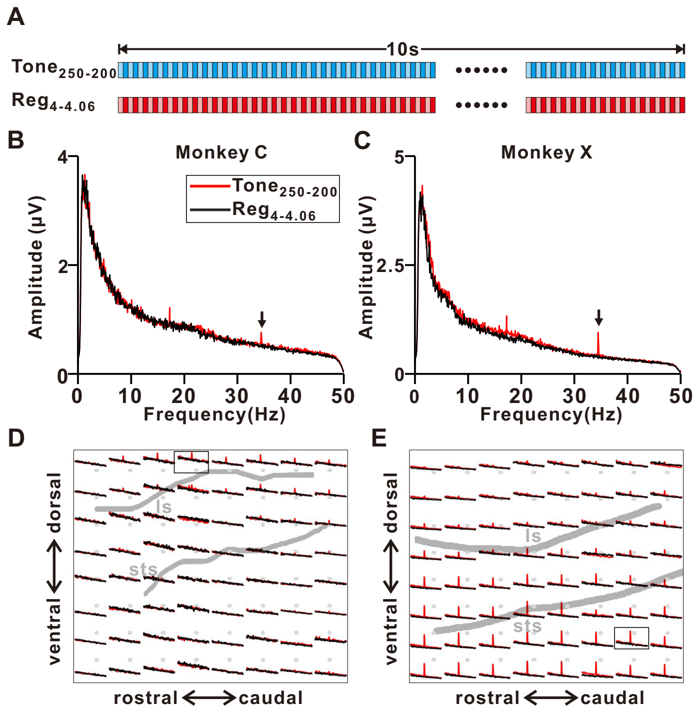

Supplementary Figure 11

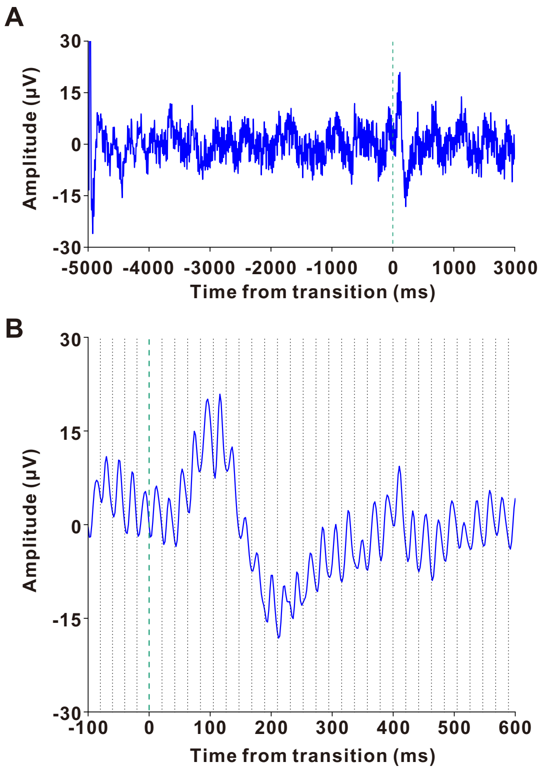

Supplementary Figure 12

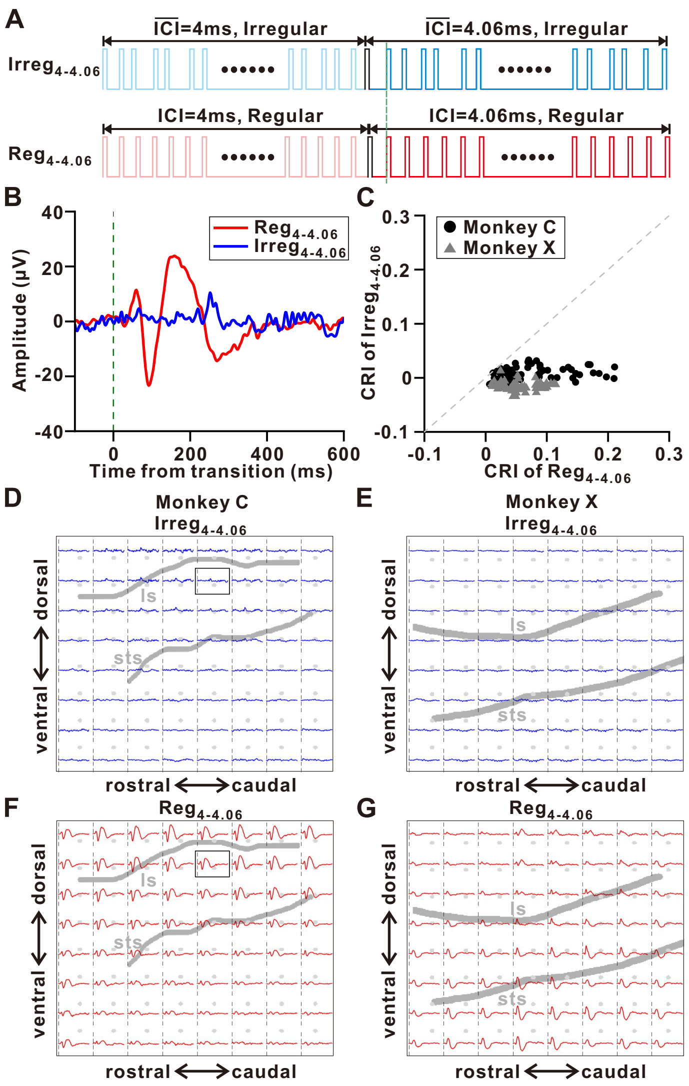

Supplementary Figure 13

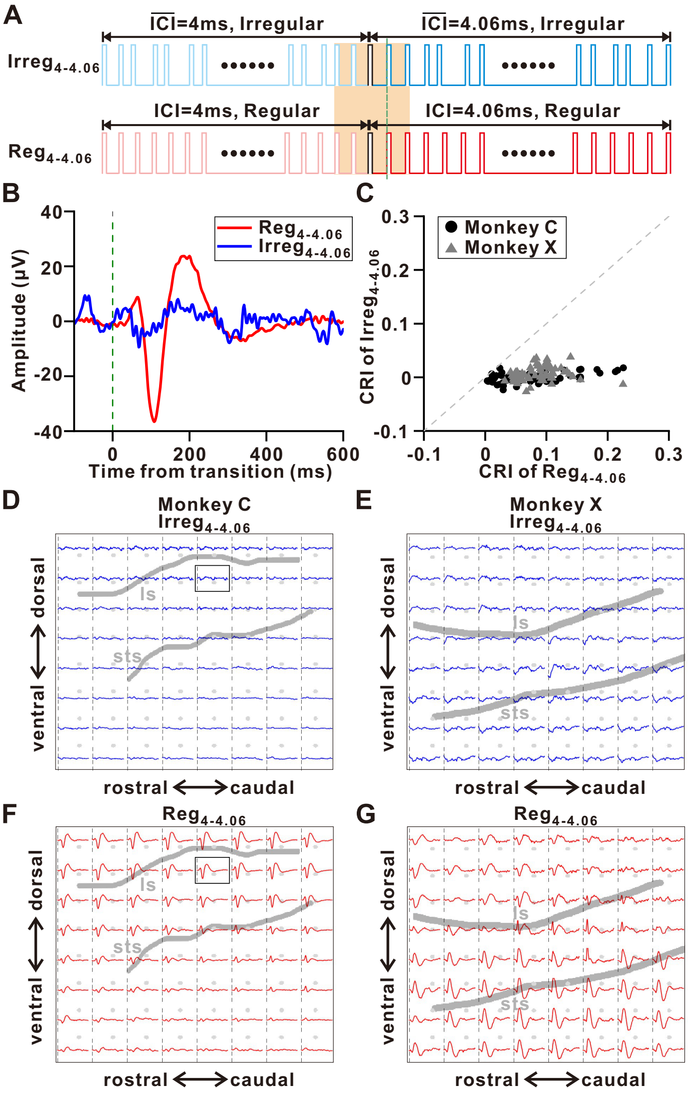

Supplementary Figure 14

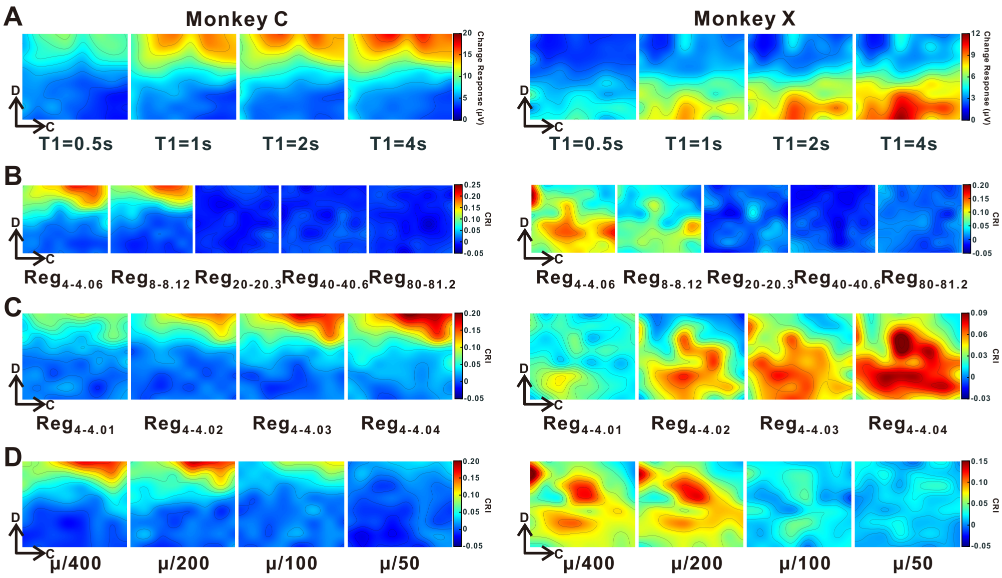

Supplementary Figure 15

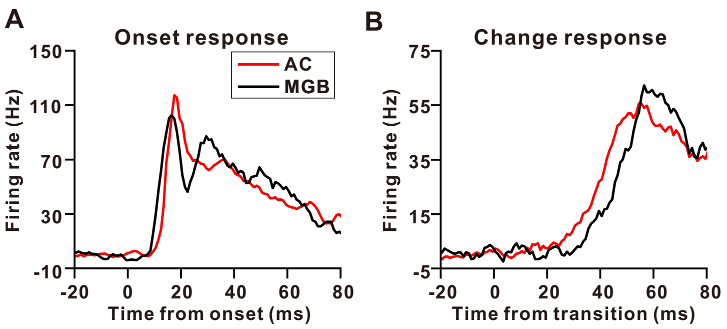

Supplementary Figure 16

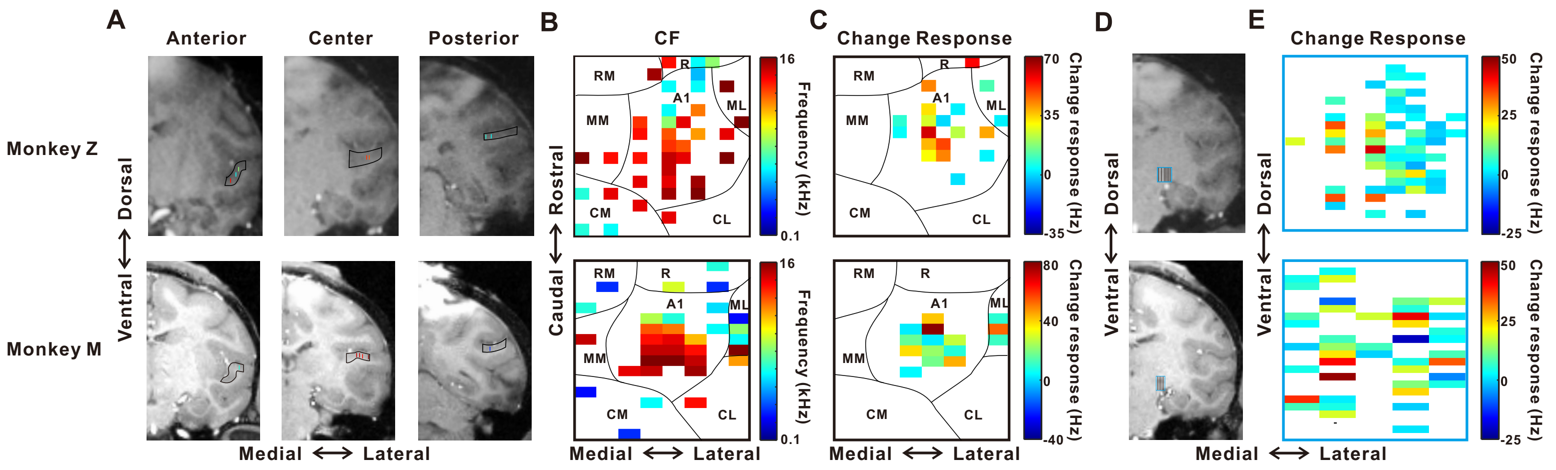

Supplementary Figure 17

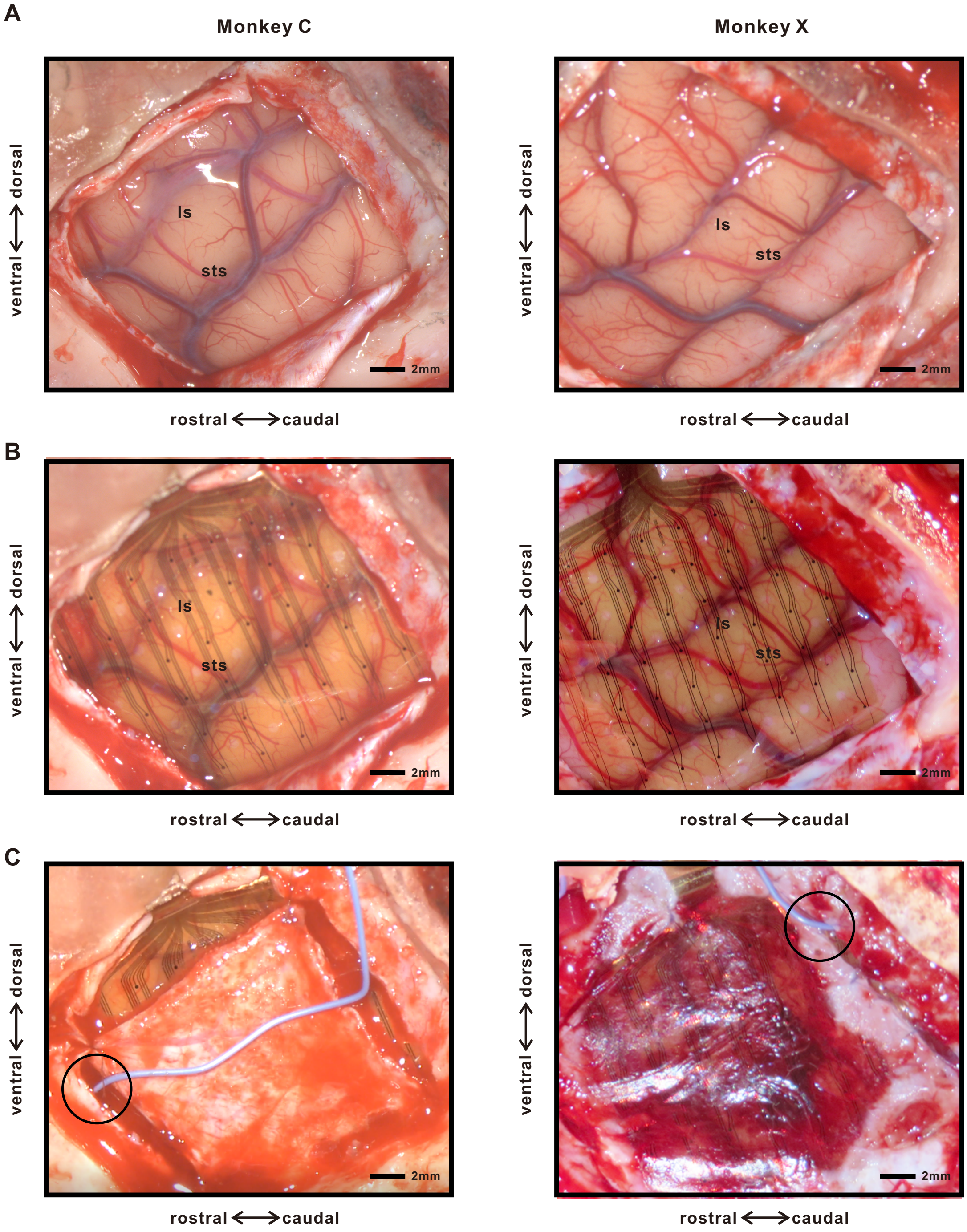

Supplement: nwaf026_Supplemental_Files [file nwaf026_supplemental_files.zip › Supplementary Figures.pdf]
