# Supplementary material for: Temporal merging into pitch with click train in the macaque auditory cortex
Source: Natl Sci Rev. 2025 Jan 22;12(6):nwaf026. doi: 10.1093/nsr/nwaf026 (PMC12139000; doi:10.1093/nsr/nwaf026)
Supplement: nwaf026_Supplemental_Files [file nwaf026_supplemental_files.zip › Supplementary Materials and Method.docx]

**Supplementary Materials and Methods**

**Subjects and Surgical Procedures**

Our research involved two male rhesus monkeys (C and X, both 7 years old, weighing 5.5–7.5 kg) for electrocorticography (ECoG) recording experiments, and two additional male rhesus monkeys (M and Z, both 8 years old, weighing 6.5–8 kg) for extracellular recording experiments. The experimental protocols strictly conformed to the guidelines approved by the State Council of the People's Republic of China (GB 14925-2010) and were sanctioned by the Bioethics Committee of Zhejiang University (ZJU20200148). This ensured the comprehensive care and ethical treatment of the animals throughout the study. Daily monitoring of the monkeys was performed by a dedicated team of researchers and animal care staff, focusing on maintaining optimal health and well-being. Enrichment strategies, including the provision of toys and preferred food items, were employed in their 0.74 m³ home cages to stimulate exploratory behaviors and improve living conditions.

For the implantation of the headpost and ECoG array, we utilized aseptic surgical techniques. Premedication involved administering ketamine (50 mg/kg) and medetomidine (0.03 mg/kg). Post-intubation, the monkeys were connected to an artificial respirator (A.D.S.1000, Engler Engineering Corp., FL) and maintained at a stable body temperature of 37°C using an electric heating mat. Continuous monitoring of vital signs, including oxygen saturation, heart rate, and end-tidal CO₂ (Surgi Vet, Smiths Medical PM Inc., London, UK), facilitated appropriate anesthesia level adjustments. The implantation process involved securing the skull in a stereotactic frame (Narishige, Tokyo, Japan). The target area was prepared post-lidocaine injection by removing skin and muscles. A titanium headpost (Gray Matter Research, MT) was affixed to the skull using resin and bone screws. Craniotomy and durotomy were performed under a microscope (Ophthalmo-Stativ S22, Carl Zeiss Inc., Oberkochen, Germany) equipped with a CMOS color camera (TS-CA-130MIII, MeCan Imaging Inc., Saitama, Japan). In Fig. S17, we provide a detailed illustration of the locations of the superior temporal sulcus (sts) and lateral sulcus (ls), as well as the electrode and reference placements, following the removal of the dura mater in both monkeys. The positional differences in the ECoG array placement may explain the variation in ECoG data observed between the two monkeys. Post-ECoG array implantation, the surgical site was sealed with the dura mater, bone flap, and skin, and the exposed skull area was covered with resin. Postoperative care involved administering ketoprofen for three days and antibiotics for one week.

In our anesthesia experiments, initial sessions were recorded with the monkeys awake. This was followed by a gradual escalation of intravenous Ketamine doses (0.5 mg/kg, 2 mg/kg, 8 mg/kg per session) while the animal was comfortably seated in a chair. Throughout the anesthesia process, continuous recordings of physiological parameters such as the electrocardiogram (ECG), heart rate, oxygen saturation, and blood pressure were meticulously conducted. Following each experimental session, a full week's rest was allotted to the monkeys before any subsequent anesthesia recording.

**Sound Stimulation**

Experiments were conducted in a sound-proof room. Acoustic stimuli were digitally generated at a 100 KHz sampling rate using a computer-controlled Auditory Workstation (RZ6, TDT) and delivered through a speaker (LS50, KEF, UK). Sound pressure levels were calibrated using a ¼-inch condenser microphone (Brüel & Kjær 4954, Nærum, Denmark) and a PHOTON/RT analyzer (Brüel & Kjær, Nærum, Denmark). Stimuli were presented contralaterally to the recording side.

We determined the frequency response area (FRA) and characteristic frequency (CF) of neuronal responses by presenting a series of tones (100 ms duration; 5-ms rise-fall time) at varying frequencies and intensities, randomized over 5 repetitions for each frequency (0.2–19.1 kHz in 26 logarithmic steps) and intensity (0–70 dB SPL in 10 dB steps), with 500 ms interstimulus intervals.

We employed two types of click trains: regular and irregular. Regular click trains consisted of pulses (0.2 ms width) with fixed intervals, while in irregular click trains (Fig. S12A, top row), pulses were presented at random intervals, following a Gaussian distribution. Three sets of irregular click trains were used in the study. The first set, shown in Fig. 3J, was designed to examine the effect of train regularity. To quantitatively assess this effect, different variances were applied to each train, including µ/400, µ/200, µ/100, and µ/50, where µ represents the mean inter-click interval (ICI) of either 4 ms or 4.06 ms. The second set, used in Fig. S8, was configured with a variance of µ/2. Likewise, the third set, presented in both Fig. S12 and Fig. S13, also employed a variance of µ/2.

Transitional click trains were formed by concatenating two 5-second regular click trains with distinct ICIs of 4 ms and 4.06 ms (Fig. 1A), marked by a shared black pulse and a green dashed transition line. Similarly, irregular transitional click trains combined two 5-second irregular click trains with different average ICIs following a Gaussian distribution with a variance of µ/2. These were labeled Reg_4-4.06_ and Irreg_4-4.06_, respectively, with audio files provided in the supplementary material. In the sound pressure level (SPL) correction experiment (Fig S7), for Reg_4-4.06_, we kept the click amplitude of Reg_4_ unchanged and scaled the amplitude of Reg_4.06_ by the square root of the ratio between 4.06 and 4. In each session, a specific stimulation was repeated 40 times, and on average, 5 sessions were conducted for each protocol.

In the human psychophysical experiment, we created a series of regular click trains of 1024 ms duration using 7 different ICI values ranging from 4 to 256 ms, following a geometric progression (Fig. S1A). Click sounds, generated under Matlab R2021a (The MathWorks, Inc., Natick, MA, USA) using a PC and sound card (Creative, AE-7) at a sampling rate of 384 kHz and 32-bit precision, were delivered via a stereo speaker (Goldern Field M23).

**ECoG Recording**

A 64-channel electrode array (HZ64-2500-50-200, NeuroNexus Technologies Inc., Michigan, USA) was subdurally implanted over the monkeys’ auditory cortex (AC) (Fig. S2A) to record surface potentials. The electrode contacts, each measuring 0.5 × 3 × 0.5 mm, consisted of gold surfaces exposed on a 2 × 2 cm polyimide substrate. Electrode impedances ranged from 20–50 kohm at 1 kHz, with an inter-electrode distance of 2.5 mm. Preoperative MRI scans guided the determination of target locations and craniotomy sizes. A gold reference electrode was positioned near the ECoG array in the subdural space, facing the dura. Lead wires from the ECoG array and reference electrodes were connected to micro connectors (ZIF-Clip 64, Tucker-Davis Technologies, TDT, Alachua, FL) in a titanium chamber affixed to the skull with resin. The ECoG signals were amplified and band-pass filtered (Butterworth, 0.5–300 Hz) using a differential amplifier (RZ5, TDT), digitally sampled at 1 kHz, and stored on hard-disk drives for subsequent analysis.

**Extracellular Recording**

In each session, we employed multi-site linear electrodes (S-probe, Plexon, USA) with 16 contacts for the AC and 32 contacts for the MGB. The electrodes were inserted into the brain guided by MRI structural imaging, passing through 26-gauge transdural guide tubes, and advanced using a remote-controlled microdrive (FHC). Neural signals were amplified 20,000-fold and bandpass-filtered between 300 Hz and 3000 Hz. Spike trains were analyzed offline using the Kilosort algorithm for spike sorting, followed by manual curation in Phy [1].

For recordings in the AC, we partitioned the regions based on the tonotopic organization of CF [2-4] . A total of 128 neurons were recorded in the primary auditory area (A1), along with 4 neurons in the rostral (R) area and 10 neurons in the mediolateral (ML) area using the transitional train stimulus. All neurons recorded in these sessions were included in the analysis. Additionally, to further refine the AC partitioning, we recorded an additional 37 neurons using pure-tone stimuli.

For the MGB recordings, we collected data from 141 neurons across a coronal cross-section spanning from the lateral to medial aspects of the MGB. Instead of targeting specific MGB subregions, we used a 32-channel linear electrode with 200-micrometer site spacing, allowing us to sample approximately 6 millimeters of depth. This approach enabled us to comprehensively record vertically across the full extent of the MGB. Tonotopic CF distributions in both the AC and MGB, as well as the distribution of change responses, are presented in Fig. S16.

**Monkey Psychophysical Experiment**

Monkeys were trained for a novelty detection task [3, 4] in two steps: initially, to press a button following a sound occurrence (usually pure tones) and subsequently, to respond only to a deviant sound. Correct responses were rewarded with a drop of water (Fig. S8A). Training sessions involved limited water access, with monkeys trained for approximately 4 hours daily. To ensure health, a minimum water intake (100-130 ml) was maintained, and their weight was monitored at least twice per week. During weekends, a full water bottle (800 ml) was provided.

Training involved pure tones in a random frequency oddball paradigm. In recording sessions, seven stimulation blocks were presented in random order, comprising three pure tone blocks, two regular click train blocks, and two irregular click train blocks. Each block, repeated 30-40 times, included control trials with standard sounds only and deviant blocks (Fig. S8B). The interstimulus interval was set at 500 ms, with each sound (including click trains) lasting 200 ms.

The behavioral protocol required monkeys to press a button within 600 ms post-deviant stimulus onset to receive a water reward. This reaction time constraint ensured decision-making wasn't based on the last stimulus in a block. In control trials, the monkey needed to refrain from pressing the button within 600 ms to receive the reward. The number of standard stimuli varied randomly between 7 to 10 in each block, preventing predictable patterns and ensuring that the monkey's response was based solely on the detection of the current deviant stimulus, rather than on counting sounds or anticipating the deviant's occurrence.

**Human Psychophysical Experiment**

In the human component of our study, 22 volunteers, aged between 20 and 30 years and all with normal hearing, participated. The study strictly adhered to ethical guidelines (IRB-20230131-R). Prior to the commencement of the experiment, informed written consent was obtained from all participants.

During the experimental sessions, participants were comfortably positioned with head support in a controlled setting, facing a keyboard and speaker. The task involved discerning gaps within a series of click trains (Fig. S1). Participants were instructed to press the right key if they detected a gap and the left key if they perceived the sound as continuous. Following the termination of each click train, a cue was presented after 800 ms, with a response window of 700 ms for the participants to register their input.

We quantified behavioral performance by plotting the proportion of 'left' choices (indicative of continuous sound perception) as a function of the ICI. Psychometric functions were fitted with a cumulative Gaussian function

$$p\left( r \right)=\frac{1}{\sigma\sqrt{2\pi}}\int_{-\infty}^{i} e^{-\frac{\left( x-\mu\right)^{2}}{2\sigma^{2}}}dx$$

Here, p(r) represents the proportion of left choices, *i* is the ICI, *μ* is the Gaussian mean, and *σ* is the standard deviation (SD). The psychophysical threshold was defined as the SD of the Gaussian fit, σ, corresponding to an 84.13% correct performance rate [5].

**Data Analysis**

The recorded ECoG signals were segmented to include a span of 3 seconds before the start and 3 seconds after the termination of each transitional click train. These signals were band-pass filtered within the 0.5–300 Hz range and subsequently down sampled to 600 Hz. Considering the different reference electrode placements in the auditory cortex of the two monkeys (Fig. S17), this may lead to variations in spatial activation patterns and ERP polarities. However, since this does not affect our experimental results, we did not perform a re-referencing procedure. To mitigate noise from non-physiological sources, we employed independent component analysis (ICA) using the FieldTrip toolbox [6].

We introduced the Change Response Index (CRI) to quantify the response magnitude of change response for each trial. CRI is defined as follows

$$CRI=\frac{RMS_{\left[ 0 300 \right]}-RMS_{\left[ -300 0 \right]}}{RMS_{\left[ 0 300 \right]}+RMS_{\left[ -300 0 \right]}}$$

Here, RMS_[-300, 0]_ refers to the root mean square (RMS) calculated from 300 ms before to the transition point (indicated by the green dashed line in Fig. 1A), and RMS_[0, 300]_ represents the RMS from 0 to 300 ms post-transition. For each condition, the CRI is typically averaged across at least 120 trials.

To compare AC responses to click train and pure tone stimuli while accounting for the possibility smaller neuronal population activated by pure tones, potentially leading to a weaker change response, we normalized the pure tone response amplitude based on the onset response (Fig. S5). The onset response was defined as the RMS within the 0-300 ms window after stimulus onset. For ECoG channels with significant onset responses to both stimuli, the pure tone response was scaled by the ratio of onset responses between the click train and pure tone. For channels lacking significant onset responses, the scaling factor was the geometric mean of the ratios from all significant channels.

For spike data, peristimulus time histograms (PSTHs) were generated using a bin width of 5 ms and a step size of 1 ms. For ease of comparison, the PSTHs of the AC and MGB were subtracted from their respective baselines, calculated over a pre-stimulus window ranging from -100 ms to stimulus onset. (Fig. 4C, G and Fig. S15).

Mirroring the methodology applied in ECoG data analysis, the Change Response Index for spike data was defined as

$$CRI=\frac{MeanFR_{\left[ 0 200 \right]}-MeanFR_{\left[ -200 0 \right]}}{MeanFR_{\left[ 0 200 \right]}+MeanFR_{\left[ -200 0 \right]}}$$

In this formula, MeanFR_[-200, 0]_ denotes the mean firing rate from 200 ms before to the transition point, while MeanFR_[0, 200]_ indicates the mean firing rate from 0 to 200 ms after the transition point.

**Statistical Analysis**

The statistical methods employed in this study primarily involved analysis of variance (ANOVA) with various factors tailored to specific analyses. One-way ANOVA was used to assess the influence of different stimulus factors on the change response within a single brain region of an individual monkey. Two-way ANOVA was applied for cross-monkey comparisons, including investigating the effect of a certain factors on the change response and neuronal response comparisons between the AC and MGB. Finally, three-way ANOVA was used during the anesthesia part to evaluate the effects of different experimental days and anesthetic depths on ECoG responses to three interval combinations for each monkey. Post-hoc tests were performed to correct for multiple comparisons using the Bonferroni method.

Furthermore, t-tests were used to assess the presence of significant change responses under specific stimuli and during the Fast Fourier Transform (FFT) analysis of neuronal oscillations at specific switching rates.

The Chi-squared test was applied to analyze behavioral experiment results from each session involving monkeys and to compare the proportion of neurons exhibiting a change response between the AC and MGB (Supplementary Table 1). To evaluate the significant effect of train regularity on novelty detection behavior across sessions, the Wilcoxon rank-sum test was performed for each monkey.

**References**

1. Pachitariu M, Steinmetz N, Kadir S *et al.* Kilosort: realtime spike-sorting for extracellular electrophysiology with hundreds of channels. *BioRxiv*. 2016: 061481.

2. Song P, Xu H, Ye H *et al.* A new function of offset response in the primate auditory cortex: marker of temporal integration. *Commun Biol*. 2024; **7**(1): 1350. doi: 10.1038/s42003-024-07058-9

3. Du X, Xu H, Song P *et al.* Beyond Auditory Relay: Dissecting the Inferior Colliculus’s Role in Sensory Prediction, Cognitive Decision-Making, and Reward Prediction. *eLife*. 2024; **13**.

4. Gong Y, Song P, Du X *et al.* Neural correlates of novelty detection in the primary auditory cortex of behaving monkeys. *Cell Rep*. 2024; **43**(3): 113864. doi: 10.1016/j.celrep.2024.113864

5. Yu X-j, Dickman JD, DeAngelis GC *et al.* Neuronal thresholds and choice-related activity of otolith afferent fibers during heading perception. *Proceedings of the National Academy of Sciences of the United States of America*. 2015; **112**(20): 6467-6472. doi: 10.1073/pnas.1507402112

6. Oostenveld R, Fries P, Maris E *et al.* FieldTrip: open source software for advanced analysis of MEG, EEG, and invasive electrophysiological data. *Computational intelligence and neuroscience*. 2011; **2011**: 1-9.
