# Supplementary material for: Temporal merging into pitch with click train in the macaque auditory cortex
Source: Natl Sci Rev. 2025 Jan 22;12(6):nwaf026. doi: 10.1093/nsr/nwaf026 (PMC12139000; doi:10.1093/nsr/nwaf026)
Supplement: nwaf026_Supplemental_Files [file nwaf026_supplemental_files.zip › Supplementary Table.docx]

**Supplementary Table**

Table 1. Comparison between the MGB and AC in terms of the percentage of neurons with significant responses to transitional trains.

|  | ICI contrast | | | | ICI length | | |
| --- | --- | --- | --- | --- | --- | --- | --- |
|  | Reg_4-4.01_ | Reg_4-4.02_ | Reg_4-4.03_ | Reg_4-4.04_ | Reg_4-4.06_ | Reg_8-8.12_ | Reg_16-16.24_ |
| AC | 33/142 | 47/142 | 57/142 | 68/142 | 82/142 | 19/142 | 4/142 |
| MGB | 4/141 | 14/141 | 10/141 | 30/141 | 49/141 | 9/141 | 0/141 |
| Chi-square test | 3.57e-7 | 2.14e-6 | 6.18e-11 | 2.55e-6 | 5.54e-5 | 0.009 | 0.048 |
